# A global meta‐analysis reveals contrasting impacts of air, light, and noise pollution on pollination

**DOI:** 10.1002/ece3.9990

**Published:** 2023-04-18

**Authors:** Solène Guenat, Martin Dallimer

**Affiliations:** ^1^ Sustainability Research Institute, School of Earth and Environment University of Leeds LS2 9JT Leeds UK; ^2^ Institute of Landscape Planning and Ecology University of Stuttgart Keplerstraße 11 D‐70174 Stuttgart Germany; ^3^ Swiss Federal Research Institute for Forest Snow and Landscape WSL Zürcherstrasse 111 8903 Birmensdorf Switzerland

**Keywords:** anthropogenic disturbances, ecosystem functions and services, plant reproductive success, pollinators, pollutants, synthesis

## Abstract

In the face of biodiversity decline, understanding the impact of anthropogenic disturbances on ecosystem functions is critical for mitigation. Elevated levels of pollution are a major threat to biodiversity, yet there is no synthesis of their impact on many of the major ecosystem functions, including pollination. This ecosystem function is both particularly vulnerable as it depends on the fine‐tuned interaction between plants and pollinators and hugely important as it underpins the flora of most habitats as well as food production. Here, we untangle the impact of air, light, and noise pollution on the pollination system by systematically evaluating and synthesizing the published evidence via a meta‐analysis. We identified 58 peer‐reviewed articles from three databases. Mixed‐effects meta‐regression models indicated that air pollution negatively impacts pollination. However, there was no effect of light pollution, despite previous studies that concentrated solely on pollinators suggesting a negative impact. Evidence for noise pollution was extremely limited. Unless action is taken to tackle air pollution, the capacity to support well‐functioning diverse pollination systems will be compromised, with negative consequences for habitat conservation and food security.

## Graphical abstract

Pollination is a key ecosystem function, yet there is no synthesis of how it is impacted by global changes such as increases in air, light, and noise pollution. With this meta‐analysis of 58 peer‐reviewed articles, we show that air pollution negatively impacts pollination, but detect no effect of light pollution and identify a lack of evidence regarding the impact of noise pollution on pollination.

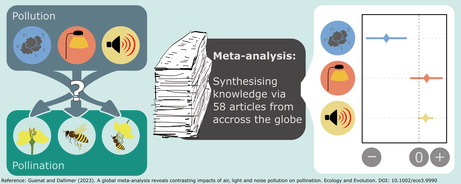

## 1 INTRODUCTION

Among the greatest challenges facing humanity in the coming decades is the need to reverse biodiversity losses that are driven by the detrimental effects of anthropogenic disturbances. Some of the most prominent threats to biodiversity include elevated levels of certain pollutants such as various air pollutants (Defra, 2019), artificial light (Hölker et al., 2010; Kyba, 2018), and noise (EEA, 2020). Sources of these pollutants are diverse and include transport, industry, energy generation, shipping, and agriculture (Defra, 2019; Hölker et al., 2010). In addition, light pollution, often seen as “sky glow,” is generated by lit buildings, streets, and security lights (Longcore & Rich, 2004). As more than 70% of land area is affected by road‐induced air, light, and noise pollution (Phillips et al., 2021), aside from the most remote areas, the impacts of pollution will be felt across virtually all habitats and ecosystems.

The impacts of pollution on organisms are known to be wide‐ranging. For plants, air pollution can decrease the sugar and water content of flowers, modify phenology (Kumar Rai, 2016), increase forest carbon content (Paoletti et al., 2010), and decrease overall diversity and abundances of herbaceous vegetation (Clark & Tilman, 2008). Light pollution impacts many organisms, for instance by changing the biological timings, including daily and seasonal activity patterns, of birds and zooplankton (Gaston et al., 2017) or the hormone levels, daily activity patterns and reproductive success of rodents and birds (Sanders et al., 2021). Noise pollution is perhaps less studied, but can impact all taxonomic groups of animals (Kunc & Schmidt, 2019) through changes in reproductive fitness, community interactions (Shannon et al., 2016) or modifications at the cellular and molecular level (Kight & Swaddle, 2011). The detrimental impact of noise on bird population densities and interference with communication is widely documented (Kociolek et al., 2011). Additionally, the hearing range of invertebrates overlaps with frequencies of common sources of anthropogenic noise (Morley et al., 2013), suggesting that impacts may be underexplored.

A main goal of conservation is the maintenance of functioning ecosystems. One of the key ecosystem functions is pollination, which contributes to supporting the reproduction of vegetation thereby securing viable habitats and underpinning food production. Pollination itself is defined as “the transfer of pollen from an anther to a stigma[, occurring] within flowers of the same plant, between flowers of the same plant, or between flowers of different plants (or combinations thereof)” (IPBES et al., 2019, p. 487) and is essential for plant reproduction. It can be either wind, water or animal mediated. Its outcome is dependent on the physiological ability of plants to produce fruits with viable seeds and, for animal‐pollinated plants, on both the status of the pollinator population and its ability to interact with the plant. Pollination as an ecosystem function is currently under threat, and declines in pollinators across all landscapes have been recorded (IPBES et al., 2019; Potts et al., 2010). This is critical as pollinators facilitate reproduction of 87.5% of wild plants (Ollerton et al., 2011) and three quarters of the world's food crops (Klein et al., 2007).

One identified driver of pollinator declines is sensitivity to various types of environmental pollution (IPBES et al., 2019). However, most evidence on the subject is derived from agricultural landscapes, synthetic pesticides/fertilizers, and heavy metals (IPBES et al., 2019). Nevertheless, we might expect there to be considerable impacts of air, noise, and light pollution on pollinators.

Air pollution has been suspected to have a deadly impact on pollinators since the early 1900s (Doane, 1917). Typically, air pollution can diminish memory and learning capacities of pollinators (Leonard et al., 2019) or negatively affect their heart rates, immunity, and resistance to stress (Thimmegowda et al., 2020). Additionally, air pollution is known to impact plants, including lowering species richness of plant communities (Zvereva et al., 2008), causing foliar injuries to herbaceous vegetation, reducing tree growth, or decreasing resilience to other stress factors (Grulke & Heath, 2020). However, little work covers plant reproduction, despite the fact that we know that air pollution can degrade pollen through alterations to the chemical structure, physical form, and abundance (Choël & Visez, 2019). Similarly, how interactions between plants and pollinators are altered by pollution is poorly known. Nevertheless, we might expect there to be negative impacts. Plant volatiles are known to be influenced by air pollution (McFrederick et al., 2009). Specifically, air pollution can alter and degrade the plant volatile compounds, thereby altering how plants interact with insects (Jamieson et al., 2017), or induce a physiological stress which might reduce the ability of plants to produce volatiles (Jürgens & Bischoff, 2017). Additionally, anthropogenic volatiles that are characteristic of air pollution might impede the ability of insects to identify flower volatiles (Jürgens & Bischoff, 2017).

The light–dark cycle is now substantially modified compared with preindustrial patterns (Irwin, 2018). Associated impacts of light pollution on insect behavior have received some research attention. Light disturbance leads to (i) the modification of visual cues necessary for orientation and foraging of nocturnal insects (Grubisic et al., 2018; Owens et al., 2020); (ii) attracts insects to lamp sources where they either become prey (Macgregor et al., 2015; Owens et al., 2020) or get killed by touching the warm lamps (Grubisic et al., 2018), or (iii) lowers reproduction due to suppressed oviposition (Macgregor et al., 2015). Work on pollinators has concentrated on moths (Grubisic et al., 2018; Macgregor et al., 2015; Owens et al., 2020). Findings tend to corroborate what we know of the impact of light on other insects. Studies on the impact of light pollution on plants are scarce in general and tend to investigate physiological aspects such as leaf retention (Bennie et al., 2016) or primary production (Singhal et al., 2019). However, while air and noise pollution can be expected to have largely negative impacts, light pollution can benefit some organisms, for example, by extending flowering time or enhancing plant growth (Bennie et al., 2016). Such differing impacts depending on the organisms could lead to knock‐on effects in which the pollination function is disrupted. Additionally, the interactions between plants and pollinators are likely to be influenced by light pollution, such as when the foraging behavior of insects is disturbed by altered light patterns (Grubisic et al., 2018). Finally, should light pollution induce seasonal and/or daily shifts of activity in pollinators (Owens et al., 2020) or phenological shifts in plant flowering time (Bennie et al., 2016), resulting temporal mismatches might lead to a collapse of animal pollination.

Noise impacts on birds are well established (Kociolek et al., 2011), raising the possibility that the 900 species of bird that are known to contribute to pollination (Nabhan & Buchmann, 1997) may suffer adverse effects. Despite the overlap between insect hearing and noise pollution frequencies, we remain almost entirely unsure of noise effects on invertebrates, the largest group of pollinators, as studies on noise pollution rarely focus on invertebrates (Kunc & Schmidt, 2019; Shannon et al., 2016), which encompass the largest group of pollinators. Noise is also known to affect plants and can alter physiology, behavior, and gene expression (Bhandawat & Jayaswall, 2022), but whether impacts extend to plant reproduction is unknown.

The need for a better understanding of how air, light, and noise pollution affects pollination is well established (Macgregor et al., 2015; McFrederick et al., 2009; Owens et al., 2020). However, how pollution interferes with pollination is so understudied that, although identified as a major threat (Balvanera et al., 2019; IPBES et al., 2019), we still lack any quantitative review synthesizing the existing research across all three types of pollution and the whole pollination system. Here, we ask whether air, light, and noise pollution has an impact on the pollination system, as defined by its three sections, namely (1) plant reproductive success, (2) pollinators, and (3) interactions between plants and pollinators. We also ask whether such impacts differ according to (a) the pollutants themselves, (b) the section of the pollination system investigated, (c) the proxies used for pollination, (d) the organisms involved, (e) the habitats investigated, or (f) the study designs.

## 2 METHODS

We carried out a review of the literature to map the extent of published evidence from 1900 onwards to assess the impact of air, light, and noise pollution on pollination. We synthesized the available evidence through a meta‐analysis.

### 2.1 Literature review

We followed the Preferred Reporting Items for Systematic Reviews and Meta‐Analysis (PRISMA), for which we developed a review protocol (Appendix S1–S3) defining the eligibility criteria, the information sources and search strategy, the study selection and data collection processes, the data items to extract, and the data analysis process (Moher et al., 2009; Page et al., 2021).

### 2.2 Eligibility criteria

To be included in the review, the articles had to report, in English, French, Spanish, or German, on original research published in a peer‐reviewed journal. Any gray literature, commentary, or articles that synthesized other research were excluded. We acknowledge that this might misrepresent the summary of the effect sizes by excluding literature more likely to contain statistically nonsignificant results (Konno & Pullin, 2020). We did not exclude studies based on simulations as those are increasingly perceived as experimental systems in their own rights (MacPherson & Gras, 2016).

Studies needed to assess the impact of air, light, or noise pollution on the pollination system. We defined air pollution as the release of substances (pollutants) in the air with direct harmful impacts on health and the environment. We used the list of pollutants included in Defra's Clean Air Strategy (Defra, 2019). This excludes soil contaminants, dusts, insecticides, and pesticides (Defra, 2019). Light pollution was defined as resulting from direct glare, consistently increased illuminations, or fluctuation of the light intensity (Longcore & Rich, 2004). It can include light resulting from anything from lighted buildings to undersea research vessels and can include changes in illumination and spectral content, including ultraviolets (Longcore & Rich, 2004). Noise pollution was defined as any anthropogenic noise, arising as a result of, for example, transport, industrial activities, or urban areas (Sordello et al., 2019). The pollination system was defined by its three sections, namely plant reproductive success, the pollinators, and the interactions between plants and pollinators. Plant reproductive success, measured through proxies such as seed and fruit set, constitutes the outcome of the pollination function. It can be measured whether the plant is wind‐ or animal‐pollinated. Pollinators represent the vectors necessary, for animal‐pollinated plants, for pollination to take place (IPBES et al., 2019). We included known pollinator taxa, based on Abrol (2012) and IPBES et al. (2019). Measures of pollinators can include community metrics (e.g., abundance and diversity), behaviors (e.g., proboscis responses to olfactory cues), physiological responses (e.g., resistance to heat stress), or morphological adaptations. The interaction between plants and pollinators includes both directly observable interactions such as pollinator visitation rates and indirect measurements such as the amount of pollen transferred or the extent to which pollinators are attracted by plant volatiles. Specific search terms and search strings can be found in Appendix S2 and S3.

### 2.3 Information sources

We included three electronic databases covering environmental and agricultural sciences, namely the Web of Science Core collection (from 1900), CAB Abstracts (from 1973), and Scopus (from 1966). Searching was carried out on July 5, 2022. Reference lists from review articles extracted from the initial search were also screened and relevant articles included.

### 2.4 Selection process

A two‐stage screening process, carried out independently by two researchers, was used to select the studies. The first stage consisted of screening the title and abstract of the complete database and excluding those not fitting the eligibility criteria. The second stage consisted of screening the full text. Full texts were excluded when the subject did not align with the eligibility criteria or if the data were not available. Overall, the two researchers had a 93% degree of agreement. In either step, articles classified differently by each researcher were discussed until consensus was reached. No assessment or scoring of the robustness of each study was however carried out, which can have implications for the robustness of the synthesis.

### 2.5 Data extraction

We extracted background information from each article. Background information included the publication year, objectives, study design (namely the “set of methods and procedures used to collect and analyse data on variables specified in a particular research problem” (Ranganathan & Aggarwal, 2018, p. 184), here either cross‐sectional, longitudinal, experimental, or simulation modeling), type of pollution (air, light, and noise) and specific pollutants, section of the pollination system studied, pollination measures used for each experiment and/or observation, country of study, target habitat, and organism(s).

For the meta‐analysis, we extracted data from each study, be that an experiment or observation, reported in each article. Data extracted included either (i) sample sizes, means and standard deviations of the two contrasting conditions (polluted and not); or (ii) the sample sizes and percentage change. Data were extracted from the text, tables, supplementary materials or, with WebPlotDigitizer v4.4 (Rohatgi, 2020), from figures.

### 2.6 Synthesis

We used a meta‐analysis to synthesize the impact of pollution on pollination. Meta‐analyses provide estimates of the effects across subjects (Koricheva & Gurevitch, 2013), allow for the understanding of whether an effect varies between studies, and identify any factors influencing this variation.

We used the sample sizes, means, and standard deviations to calculate Hedges' g; a common metric to estimate the standardized difference among means that is unaffected by different sampling variances across group and corrects for small sample sizes (Rosenberg et al., 2013). When results were presented as a proportion of change from the polluted to the nonpolluted state rather than with the mean and standard deviations, we used the sample sizes and proportion of change to calculate the log odds' ratio, which we then converted to Hedges' g (Borenstein et al., 2009). For articles containing multiple studies with different subgroups that were not relevant for the aims of our review, we combined the data for the different subgroups to compute an overall effect size and variance for the whole study by following the procedure described in Borenstein et al. (2009). We kept the data separate in cases where the experiments and/or observations differed in regard to the pollutants, the section of the pollination system, the pollination measures, the study design, the habitat investigated, and/or the organisms (defined as wind‐ or animal‐pollinated for plants and order level for pollinators).

We analyzed the data with two model selection processes, one for air and one for light pollution, and a random‐effects model for noise pollution. For all models, we used Hedges' g as the response variable. For the model selection processes, we ran two mixed‐effects meta‐regression models, one each for air and light pollution, with the following six explanatory variables, known as moderators in meta‐analysis: the section of the pollination system investigated, the pollutants, the pollination measure, the study design, the habitat investigated, and the organisms. As is normal practice for meta‐analyses, we weighted the studies by the inverse of their sampling variance. We used the Akaike Information Criteria with correction for small sample size (AICc) to compare models with all the potential combinations of the six moderators. Models with a ΔAIC_c_ ≤ 2 were selected (Burnham & Anderson, 2002). We then carried out subgroup analysis for all variables selected within the best‐fit models to investigate causes of heterogeneity. For the noise model, we decided to not add moderators to the random‐effects model, as noise pollution was only studied in three articles. We thus used a random‐effect model, with Hedges' g as response variable, but without any explanatory variables, and we weighted the studies by the inverse of their variance. We tested for publication bias by visually exploring funnel plots and carrying out Eggers' regression test to all best‐fit models (Egger et al., 1997) and adjusted the analysis with a trim and fill correction for the best‐fitting models on light and noise pollution, in which such bias was identified (Shi & Lin, 2019). All analyses were carried out in R v.4.1.2 (R Core Team, 2021), with the metafor package for effect size and model calculations (Viechtbauer, 2010) and the MuMIn package for model selection (Barton, 2020).

## 3 RESULTS

### 3.1 Extent of the knowledge

**FIGURE 1:**
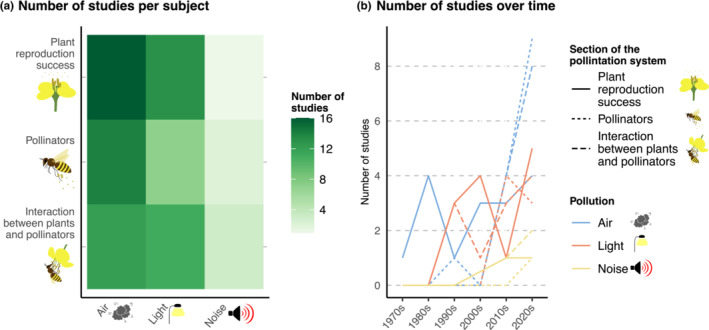
Scope of the studies investigated. (a) Number of studies on the impact of air, light, and noise pollution on sections of the pollination system (plant reproductive success; pollinators or interactions between plants and pollinators) and (b) number of studies over time.

Of the 2192 articles that were initially extracted, 58 were relevant to the impact of pollution on the pollination system (Appendix S4; Figure 1a). Articles included up to 18 different studies (median = 1). Most articles (*n* = 33) focused on the impact of air pollution, followed by light (*n* = 22) and just four on noise pollution. Only one article investigated two types of pollution concomitantly, namely light and noise. Proxies for pollination were nearly equally spread out within the three sections of the pollinations systems, with 29 articles related to plant reproductive success, 21 related to pollinators, and 27 to the interaction between plant and pollinators. Most articles included several observations, with 17 articles using multiple proxies relating to different sections of the pollination system: six focusing both on interactions between plants and pollinators and on plant reproductive success, seven focusing on both pollinators and the interaction between plants and pollinators and two focusing on both plant reproductive success and pollinators. Two articles used proxies pertaining to all three sections of the pollination system (Figure 1a, Appendix S4).

The distribution of studies across the different types of pollution and sections of the pollination system was uneven. Among the studies on the impact of air pollution, 16, 12, and 14 investigated how it affects plant reproductive success, pollinators and the interaction between plants and pollinators, respectively (Figure 1a, Appendix S4). There was less balance in terms of which sections of the pollination system were studied in relation to light pollution, with 13 studies investigating plant reproductive success, seven the pollinators and eleven the interactions between plants and pollinators. The little we know about the impact of noise pollution on pollination comes from four studies, one focusing on pollinators, one on the interaction between plants and pollinators and one on both plant reproductive success and the interaction between plants and pollinators.

The number of published articles on the impact of pollution on pollination has been relatively consistent between the 1980s and the 2000s, with a sharp increase from the 2010s, and an even sharper increase in the 2020s (Figure 1b). Earlier articles focused solely on the impact of air pollution on plant reproductive success (Figure 1b), with most articles coming from the agricultural sciences. Articles focusing on the impact of pollution on the animal side of the pollination system, either through pollinators or through the interaction between plants and pollinators, started in the late 1990s and grew steadily (Figure 1b).

**FIGURE 2:**
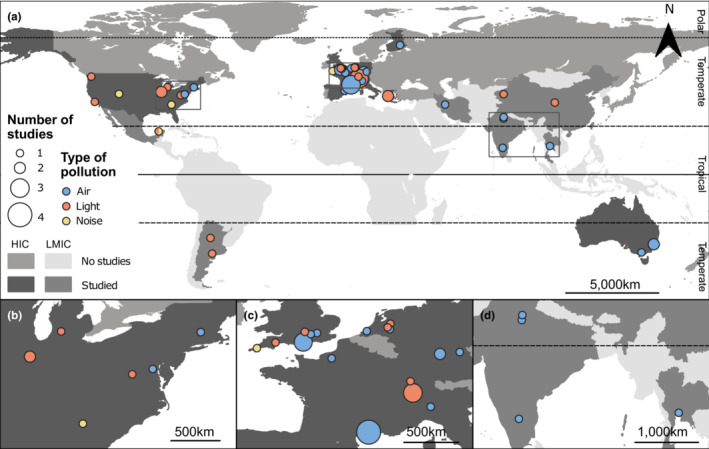
Geographical distribution of the 58 studies found on air (*n* = 33), light (*n* = 22), and noise (*n* = 3) pollution. LMIC—low‐ and middle‐income countries; HIC—high‐income countries. (a) Worldwide; (b) North America's East Coast; (c) Central Europe; (d) Southeast Asia.

Studies were conducted in 17 countries, with 83% of all studies carried out in high‐income countries (Figure 2). Most took place in temperate regions, with only three focusing on tropical regions (Figure 2).

### 3.2 Study designs

Four different study designs were used, with experimental (*n* = 46) being the most common, followed by cross‐sectional (*n* = 9), simulations (*n* = 2) and longitudinal (*n* = 1) studies (Appendix S5). Experimental studies were mainly carried out in laboratories and/or greenhouses (*n* = 29) but also included 17 field experiments. Laboratory and field experiments about air pollution differed by the concentration of pollutants. For example, the difference in ozone concentrations between the controls and the treatments was of an average (±SE) of 173 ppb (±30.5) in laboratory experiments, as opposed to 38.75 ppb (±13.05) in field experiments. Such differences between laboratory and field experiments were less obvious for light pollution. UV‐B averaged 7.35 kJ (±1.7) and 6.45 kJ (±2.2) for laboratory and field experiments, respectively. Outdoor habitats investigated, through either the 17 field experiments or the seven cross‐sectional studies, included grasslands (*n* = 6), agricultural landscapes (*n* = 7), forests (*n* = 5), urban areas (*n* = 8), and deserts (*n* = 1) (Appendix S6).

### 3.3 Pollution

**FIGURE 3:**
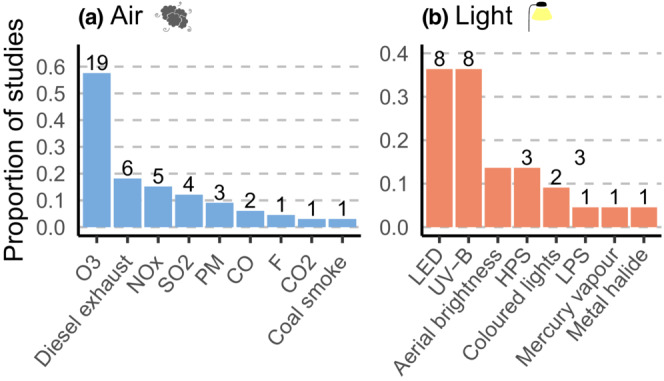
Pollutants investigated by articles on (a) air pollution (*n* = 33) and (b) light pollution (*n* = 22). Bars indicate the proportion of studies investigating the specific pollutant out of all the studies focusing on either air or light pollution, and values indicate the number of articles. Articles in which pollutants were not specified were excluded (*n* = 1 for air, *n* = 0 for light).

Within the 33 articles on air pollution, the impact of 10 different pollutants was investigated, with a median number of one per article, though six articles investigated a mixture of two to six different pollutants (Figure 3; Appendix S7). The most commonly studied pollutant was ozone (*n* = 19), followed by diesel exhaust (*n* = 6), nitrogen oxides (*n* = 5), and sulfur dioxide (*n* = 4) (Figure 3, Appendix S7). There were eight different light pollutants studied, with UV‐B (*n* = 8) and LED lights (*n* = 8 each) being the most investigated, followed by high‐pressure sodium street lamps (*n* = 3), colored lamps, and illumination (*n* = 2 each; Figure 3, Appendix S7). All but two studies investigated only one type of light pollutant, with the exceptions studying five and two types of light pollutant, respectively (Appendix S7). One study on noise specifically investigated traffic noise, while the others did not differentiate between different types of noise.

### 3.4 Pollination

The measures of pollination were diverse across all the sections of the pollination system, and studies used up to six different proxies to measure pollination (median = 1, Appendix S8). Plant reproductive success was measured with up to four different proxies per article (median = 1; Appendix S8). These included the proportion of flowers fruiting (*n* = 17), the number of seeds per fruit (*n* = 12), fruits per plant (*n* = 8), seeds per plant (*n* = 5), the mass of seeds per plant (*n* = 2), or the pollinators' contribution to crop production (differences in seed number with exclusion cages, *n* = 1; Appendix S8). Up to three different proxies per article were used to assess the impact of pollution on pollinators (median = 1, Appendix S8). Proxies were related to community indicators, such as abundance (*n* = 7) and species richness (*n* = 2), to behaviors, with the most common proxies linked to motility (*n* = 7) and to the proboscis responses to olfactory cues (*n* = 5), to physiological responses (*n* = 4) such as resistance to heat stress or heart rates, or to morphological adaptations (*n* = 2) such as wing symmetry. A maximum of two different methods (median = 1) were used to measure the interaction between plants and pollinators (Appendix S8). The measures included the number (*n* = 11) and length (*n* = 4) of pollinator visits, the number of flowers visited (*n* = 5), changes in feeding behavior (*n* = 3), various proxies for pollen transfer (*n* = 4), as well as changes in the quantity or attractiveness of plant volatiles (*n* = 3) and the perception of such volatiles (*n* = 7).

Across all articles, 52 plant species were investigated for the effect of pollution either on their reproductive success (*n* = 21), on the interaction between plants and pollinators (*n* = 24), on both plant reproductive success and interactions between plants and pollinators (*n* = 6), or to study pollinators' behavioral responses to their scent (*n* = 1). Of the 52 plant species, two are solely wind‐pollinated while the other 50 rely to some extent on animal pollination (Appendix S9). Studies investigated up to 20 different plant species (median = 1). The only plant species investigated in more than two articles were *Brassica napus* (rapeseed, *n* = 5) and *B. nigra* (black mustard, *n* = 4).

Individual pollinator species included in experiments were mostly bees (honeybees *n* = 11, bumblebee species *n* = 7, mason bees *n* = 1) and Lepidoptera (moths = 9, with seven different species, butterflies *n* = 2). Fly and beetle pollination was investigated in six and two articles, respectively, and birds were covered in a single article (Appendix S10).

### 3.5 Impact of pollution

**FIGURE 4:**
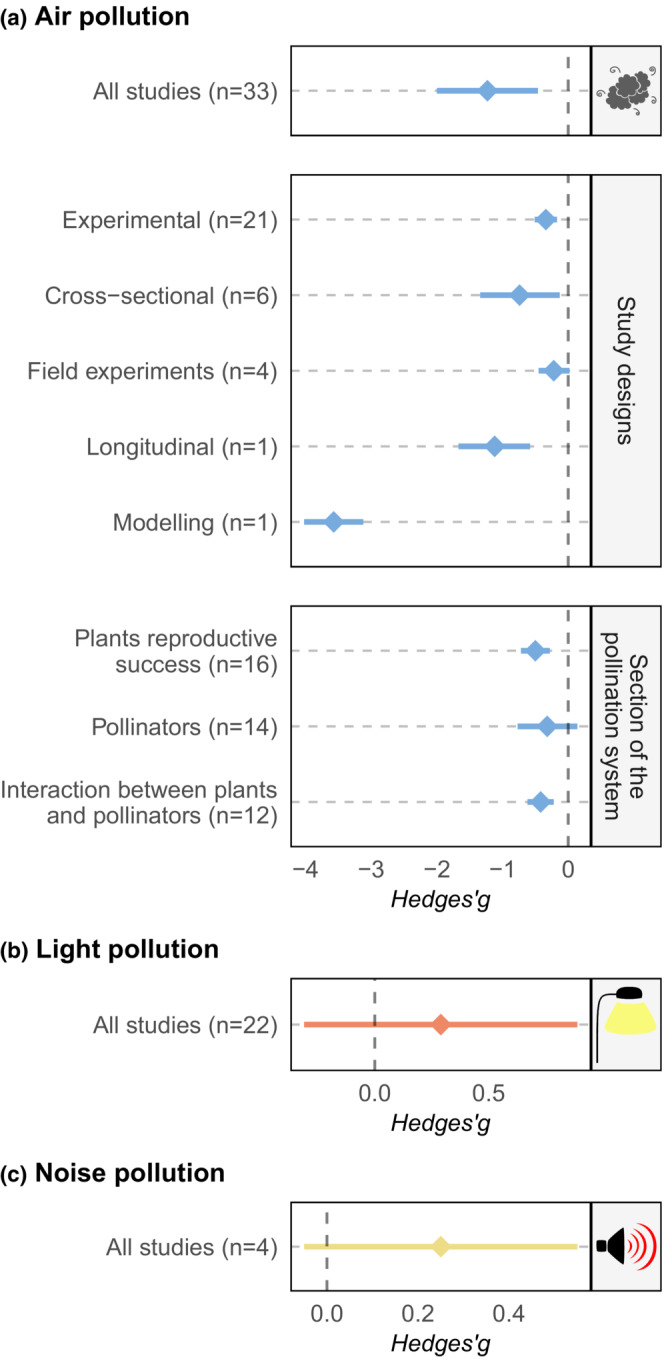
Effect sizes and 95% confidence intervals of the overall models for air, light, and noise pollution, and for the subgroup models for each variable in the best‐fit models. Diamonds represent the effect size, and bars represent the confidence intervals. Effects are considered significant when the confidence intervals do not cross the zero line.

Air pollution had a significant impact in decreasing pollination (*μ* = −1.23; 95% CI = −2.00, −0.46; *p* < .001; Figure 4a and Appendix S11 and S12). The two moderators selected as contributing to the single best‐fit model were the section of the pollination system and the study design (Appendix S11 and S12), which had a significant impact on findings (QM test of moderators: QM = 49.66, *p* < .01). Submodels showed that studies with cross‐sectional (*μ* = −0.74; 95% CI = −1.34, −0.14; *p* = .017; Figure 4a), experimental (*μ* = −0.34; 95% CI = −0.51, 0.17; *p* < .001; Figure 4a), simulations (*μ* = −3.57; 95% CI = −4.02, −3.12; *p* < .001; Figure 4a), or longitudinal (*μ* = −1.12; 95% CI = −1.67, −0.58; *p* < .001; Figure 4a) designs were able to reveal a significant impact of air pollution (Figure 4a), while field experiments (*μ* = −0.22; 95% CI = −0.45, 0.02; *p* = .071; Figure 4a) did not show any impact of air pollution on pollination. Air pollution was shown to have an impact on the plant reproductive success (*μ* = −0.50; 95% CI = −0.72. ‐0.28; *p* < .001; *p* = .172; Figure 4a) and the interaction between plants and pollinators (*μ* = −0.42; 95% CI = −0.62, −0.22; *p* < .001; Figure 4a), but not on pollinators themselves (*μ* = −0.32, 95% CI = −0.77, 0.14; Figure 4a). Light pollution had no significant impact on pollination (*μ* = 0.29; 95% CI = −0.31, 0.89; *p* = .346; Figure 4b; Appendix S13 and S14). No moderators were included in the single best‐fitting model. Noise pollution had no significant impact on pollination (*μ* = 0.28; 95% CI = −0.05, 0.54; *p* = .106; Figure 4c; Appendix S15 and S16). It was not possible to investigate the effect of moderators due to the low number of studies.

## 4 DISCUSSION

We found mixed effects of air, light, and noise pollution on the pollination system. While air pollution had an overall negative effect, the impact differed according to the section of the pollination system and study design. Impacts were otherwise apparent irrespective of the measures of pollination, and different organisms and habitats investigated. In contrast, our meta‐analysis indicated that neither light nor noise pollution had any impact, although the impact of noise remains uncertain due to the low number of studies. Further, we know almost nothing about the synergistic impacts of pollutants as only a single study investigated more than one concomitantly.

Air pollution is a major cause of deaths for humans (WHO, 2016) and severely disrupts many species (Krupa, 2003; Kumar Rai, 2016). Here, we showed that it also negatively affects pollination. However, study type was an important determinant. Experimental studies tended to reveal negative impacts, whereas cross‐sectional studies and field experiments failed to reveal significant negative impacts. In part, this is likely to be due to the higher concentrations that are used in laboratory compared with field experiments or cross‐sectional studies. Additionally, there might be more context‐related variables influencing the latter two than laboratory experiments that therefore impede the identification of single processes explaining changes in pollination (Catford et al., 2022). The section of the pollination system was also important. Most evidence covered impacts on plant reproductive success, where the significant negative impact was consistent with a previous synthesis focusing on ozone only (Leisner & Ainsworth, 2012). We also found the interaction between plants and pollinators was negatively affected, but there was no evidence of significant impacts on pollinators themselves, thus hinting that the disturbances in the interaction between plants and pollinators were mediated by the reaction of plants to pollution.

Air pollution studies were scattered across disciplines, with studies from agricultural sciences (Shannon & Mulchi, 1974), forestry (Paoletti et al., 2010), and plant physiology (Linskens et al., 1985). There was however a particular focus on agricultural settings (e.g., Drogoudi & Ashmore, 2000; Ryalls et al., 2022), reflecting the fact that current research on pollination centers on its importance for food production. However, air pollution is worse in towns and cities (WHO, 2016), except for ammonia (Defra, 2019) which was not investigated by any of the identified studies. Town and cities have the potential to play a role in pollinator conservation (Baldock et al., 2015; Wenzel et al., 2020), yet we identified only three studies carried out in urban areas (Chauhan et al., 2004; Thimmegowda et al., 2020; Tommasi et al., 2022). The fact that the impact of air pollution was detected despite most studies focusing on less polluted landscapes suggests that the negative consequences for pollination may be more severe in the most polluted areas. Failure to tackle air pollution, especially in urban areas, would therefore likely contribute to the biodiversity crisis both by lowering plant reproductive success and hindering plant–pollinator interactions.

Our search of the literature also highlighted two substantial gaps in our knowledge. Both tropical regions and low‐ and middle‐income countries were understudied. These gaps are important because such regions and countries tend to experience higher levels of air pollution (WHO, 2016). Pollination is also arguably more important in the low‐ and middle‐income countries, given that, respectively, 60% and 29% of their population rely on agriculture for their livelihoods, as opposed to 3% in high‐income countries (World Bank, 2021). Further, pollination in the tropics is likely to be more susceptible than in temperate regions due to the higher proportions of plants requiring animal pollination (Ollerton et al., 2011). Work on pollinators that has been carried out in the tropics also tends to reveal differences compared with temperate regions. Pollinator networks tend to be more diverse, and vertebrate pollinators are often integral to the pollination system (Vizentin‐Bugoni et al., 2018). When anthropogenic impacts have been investigated, steeper declines in pollinator visitor rates from natural to agricultural landscapes (Ricketts et al., 2008) or variable impacts across taxa (Guenat et al., 2019) have been observed. We therefore cannot assume that trends from temperate regions will hold. While we do advocate for more research into the details, our review would support any efforts to reduce air pollution as this would ensure that negative impact on human health is not compounded by reductions in food security partly driven by declines in pollination.

Both light and noise pollution has been shown to impact some organisms by, for instance, disturbing reproduction (for moths; Macgregor et al., 2015) or altering community interactions (e.g., see Shannon et al., 2016 for a synthesis on terrestrial and marine wildlife, with most evidence from birds and marine mammals). However, our meta‐analysis of the existing evidence did not show impacts on pollination from light or noise pollution. For noise, this is likely to reflect a lack of studies, highlighting the need to further investigate the potential impacts of noise. For light, however, the lack of an effect is probably because our analyses addressed the entire pollination system, rather than individual sections. Indeed, light pollution can have both positive and negative impacts depending on the organism, as was observed in our meta‐analysis (negative effect for Altermatt and Ebert (2016), Demchik and Day (1996), Van Langevelde et al. (2017) and Wang et al. (2008); positive effect for and Collins et al. (1997) and Conner and Neumeier (2002)). Though this impedes the detection of any results in a meta‐analysis, it could be a sign of potential mismatches with negative consequences for the functioning of pollination, which depends on the precise interaction of plants and their pollinators. Similar mismatches in which some part of the pollination system is disrupted are seen in the context of urbanization, where the high proportion of exotic plant species leads to the loss of specialist pollinators (Wenzel et al., 2020). Additionally, research on light pollution thus far tends to focus on different times of the day depending on the section of the pollination system investigated. Typically, articles on pollinators tended to focus on night‐dwelling insects such as moths, studying how changes in nighttime light affected them, while those on plant reproductive success and interactions focused on daytime changes in light intensity or exposure (Petropoulou et al., 2001; Wang et al., 2008). This temporal mismatch has been shown to lead to a positive impact of increased light intensity on plant reproductive success (Feldheim & Conner, 1996; Petropoulou et al., 2001) confounding detrimental impacts of nighttime light pollution on moth populations (Boyes et al., 2021; Macgregor et al., 2015). Investigating the impact of light pollution during both day‐ and nighttime is thus critically needed. Indeed, the two studies that did so both resulted in novel insights. Plant reproductive success was decreased by light pollution but still depended on successful interactions with both nocturnal and diurnal pollinators (Knop et al., 2017), and nighttime illumination impacted diurnal plant–pollinator interactions differently according to the species (Giavi et al., 2021).

Current evidence highlights a lack of knowledge about how the effects of light pollution can be important throughout the diurnal cycle. Nighttime pollination occurs in 30% of plant families (Borges et al., 2016), and meteorological conditions such as wind, humidity, and temperature at night are different from daytime. Nocturnally pollinated plants and nocturnal pollinators tend to have specialized traits such as large floral displays, the ability to preheat flight muscles or particularly sensitive eyes (Borges et al., 2016). Nighttime light pollution may be the most prevalent type of light pollution (Longcore & Rich, 2004), but available evidence indicates that changes in light intensity or exposure during daytime can also affect plant reproductive success (Feldheim & Conner, 1996; Petropoulou et al., 2001). In parallel, flower production, which can increase pollination, tends to be improved by increased light (Kuniga, 2020). Additionally, we know that ultraviolet wavelengths are key to color reception in pollinators (Chittka et al., 1994), and so we can expect daytime changes in UV‐B to alter how pollinators interact with flowers. Consequently, drawing conclusions solely from work on nocturnal pollination systems may not capture the full extent of the impact of light pollution.

The evidence on noise pollution is extremely limited. The four studies we identified highlight the need to consider the full diversity of pollinator types and their associated life‐cycle stages. One study investigated butterflies as caterpillars, showing that short‐term exposure to highway noise increased their heart rates (Davis et al., 2018), thus indicating that potential developmental impacts could be ignored were only the adult stage studied. Despite the fact that we know birds are detrimentally impacted by noise (Kociolek et al., 2011), there was only one article covering birds as pollinators. This suggested that pollination actually increased with higher levels of noise pollution (Francis et al., 2012). Given the important role of birds as pollinators in the tropics (Sekercioglu, 2006), it will be important to determine how generalizable this finding is, and what the potential underlying mechanisms might be.

Many sources of anthropogenic pollution co‐occur (Phillips et al., 2021), and some can have co‐occurring effects, such as ozone pollution changing the properties of light. The multiplicative impact of more than one source of pollution is, therefore, both complex to study, and of potentially greater importance than one source of pollution alone. However, only one study (Dzul‐Cauich & Munguía‐Rosas, 2022) investigated both light and noise pollution. Further, pollination is known to be influenced by many different factors such as land‐use change, climate change or the introduction of non‐native species and pathogens (Potts et al., 2010; Sánchez‐Bayo & Wyckhuys, 2019). Landscape conversion through urbanization and road construction is also known to impact pollinators (Phillips et al., 2021; Wenzel et al., 2020). Though both are linked to high levels of pollution, studies rarely differentiate the impact of pollution of that of other pressures such as changes in land use. Although this review did not specifically examine the interactions between types of pollution and other anthropogenic changes, when discussed within the studies, the findings were complex. For instance, Reitmayer et al. (2019) showed that diesel exhaust reduces honeybees resistance to heat stress, a result which could compound any implications for honeybees associated with increased temperatures due to climate change or urban heat island effects (Banaszak‐Cibicka & Zmihorski, 2012). Similarly, nighttime light pollution could facilitate a temporal shift of activity that could potentially allow diurnal pollinators to adapt to the warmer temperature associated with climate change (Levy et al., 2019). This could potentially disrupt plant–pollinator interactions and reduce pollination success.

## 5 CONCLUSION

Biodiversity is subject to complex combinations of stressors, including multiple forms of pollution. However, as yet we do not fully understand the role of pollution in ecosystem functioning. Here, we synthesize the existing evidence on the impact of air, light, and noise pollution on one function, namely pollination. We highlight that air pollution negatively affects the pollination system, decreasing plant reproductive success—the outcome of pollination—and harming plant–pollinator interactions, while not affecting pollinators themselves. However, there are still substantial knowledge gaps. We have little evidence from tropical, low‐, and middle‐income countries, where air pollution levels and potential dependence of the human population on pollination‐supported food production are higher. There are also unanswered questions regarding the importance of light and noise pollution, with no detected impact by light pollution despite risks of mismatch between pollinators and plants. However, we are able to demonstrate the importance of addressing air pollution. In parallel with broadening the evidence base, our work highlights the need to lower air pollution levels if well‐functioning pollination systems are to be retained.

## AUTHOR CONTRIBUTIONS

**Solène Guenat:** Conceptualization (equal); data curation (equal); formal analysis (equal); methodology (equal); visualization (equal); writing – original draft (equal). **Martin Dallimer:** Conceptualization (equal); funding acquisition (equal); methodology (equal); project administration (equal); writing – review and editing (equal).

## Supporting information


Data S1:

## Data Availability

Data sharing is not applicable to this article as no new data were created in this study.
