# Supplementary material for: A global meta‐analysis reveals contrasting impacts of air, light, and noise pollution on pollination
Source: Ecol Evol. 2023 Apr 18;13(4):e9990. doi: 10.1002/ece3.9990 (PMC10111172; doi:10.1002/ece3.9990)
Supplement: Supplementary file 1 — Data S1: [file ECE3-13-e9990-s001.docx]

**Supplementary information for “A global meta-analysis reveals contrasting impacts of air, light and noise pollution on pollination”**

Solène Guenat^1,2,3^; Martin Dallimer^1*^

^1^ Sustainability Research Institute, School of Earth and Environment, University of Leeds, LS2 9JT Leeds, United Kingdom.

^2^ University of Stuttgart, Institute of Landscape Planning and Ecology, Keplerstraße 11, D-70174 Stuttgart, Germany.

^3^ Swiss Federal Research Institute for Forest, Snow and Landscape WSL, Zürcherstrasse 111, 8903 Birmensdorf, Switzerland.

*corresponding author: [m.dallimer@leeds.ac.uk](mailto:m.dallimer@leeds.ac.uk)

# 2. Methods


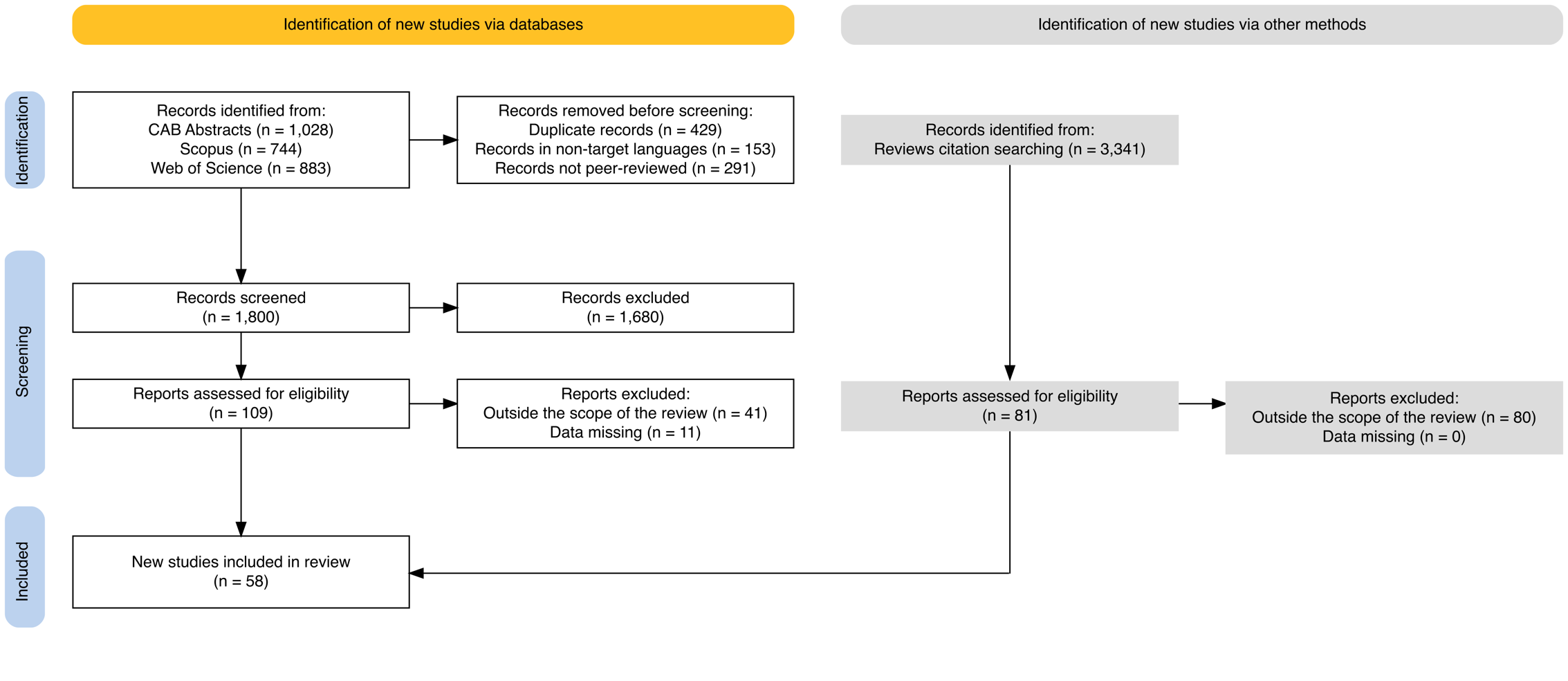


### **Appendix S1. Research process flow**, drawn on the PRISMA Flow Chart (Haddaway et al., 2022)

### **Appendix S2. List of search terms,** which were translated into search strategies for the electronic databases Web of Science, Scopus and CAB Abstracts (see Appendix S3). This table shows an abbreviated presentation of all variations of search terms. In the search, truncations, synonyms chemical symbols, and different spelling and word variants of the search terms were included as well.

| **Theme** | **Search terms** | **Source** |
| --- | --- | --- |
| Pollination | Pollination, pollinators, pollinate, reproductive success, visitation rate, fruit set, seed set |  |
| Air pollution | Air contamination, air pollutant, air pollution, air quality, atmospheric contamination, atmospheric pollution, diesel exhaust, dusts, exhaust particles, gaseous pollution, industrial pollution, ozone, O3, particulate pollution, particulate matter, persistent organic pollution, PM2.5, PM 10, road pollution, persistent organic pollutants, smog, traffic, ultrafine particles, vehicle emissions, volatile organic compounds pollution, ammonia, nitrogen oxides, nitrogen dioxides, sulphur dioxides, benzene, lead, carbon monoxides, benzo(a)pyrene, nickel, cadmium, arsenic, 1.3-butadiene, acid rain | (Curti et al., 2016; Defra, 2019) |
| Light pollution | light pollution, artificial light at night, ALAN, ultra-violet pollution, streetlight, lighted buildings, fishing boat lights, security lights, flares, light on vehicles, illuminance, skyglow | (Longcore and Rich, 2004) |
| Noise pollution | Noise, sounds, masking auditory, man-made noise, anthropogenic noise, man-made sounds, music festivals, noise pollution, transportation noise, road noise, highway noise, railway noise, motorway noise, traffic noise, urban noise, city noise, construction noise, ship noise, boat noise, port noise, aircraft noise, airplane noise, airport noise, industry noise, machinery noise, gas extraction noise, mining noise, drilling noise, pile-driving noise, communication network noise, wind farm noise, agricultural noise, farming noise, military noise, gun noise, visitor noise | (Sordello et al., 2019) |

### **Appendix S3. Search strings used with the three electronic databases.**

| Databases | Search strings |
| --- | --- |
| Web of Science | (TS=(pollinat* OR "reproductive success" OR "visitation rate*" OR "fruit* set*" OR "seed* set*") NOT TS=(freshwater OR aquatic OR fish OR *plankton* OR river OR water OR marine OR sea OR seas OR algorithm OR allerg* OR (bird* AND nest*)))  AND  ((TS=("air contamin*" OR "air pollutant*" OR "air pollution" OR "air quality" OR "atmospheric contamin*" OR "atmospheric pollut*" OR "diesel exhaust" OR (dust OR dusts) OR "exhaust part*" OR "gaseous pollut*" OR "industrial pollut*" OR "industry pollut*" OR ozone OR O3 OR (particle* AND pollut*) OR particulate* OR "particulate matter" OR "persistent organic pollut*" OR "PM2,5" OR "PM 2,5" OR "PM2.5" OR "PM 2.5" OR "PM 10" OR pm10er POPS OR "road* pollut*" OR "air pollut*" OR smog OR traffic OR "ultrafine particle*" OR "vehicle emission*" OR (VOCS AND pollut*) OR ("Volatile Organic Compounds" AND pollut*) OR (VOC AND pollut*) OR NH3 OR ammonia OR “NOx” OR “nitrogen oxid*” OR “nitrogen dioxid*” OR “sulphur dioxid*” OR benzene OR “Pb” OR “carbon monoxide” OR Benzo*pyrene OR Nickel OR Ni OR cadmium OR Cd OR Arsenic OR “1.3-butadiene” OR “1,3-butadiene” OR gaseous pollut* OR “acid rain*”) NOT TS=("particle swarm" OR algorithm OR allerg* OR neonicotinoid*))  OR  ((TI = (noise OR sound*) OR TS = ("masking auditory" OR "man-made noise" OR "anthropogenic noise" OR "man-made sound*" OR "music festival*" OR ((pollution OR transportation OR road* OR highway* OR motorway* OR railway* OR traffic OR urban OR city OR cities OR construction OR ship* OR boat* OR port* OR aircraft* OR airplane* OR airport* OR industr* OR machinery OR "gas extraction" OR mining OR drilling OR pile-driving OR "communication network*" OR "wind farm*" OR agric* OR farming OR military OR gun* OR visitor*) AND noise)) NOT TS=("ultra-sound")))  OR  (TS=("light pollut*" OR "artificial light at night" OR ALAN OR ((ultra-violet OR ultraviolet OR UV) AND pollution) OR "street light*" OR streetlight* OR "lighted building*" OR "fishing boat* light*" OR "security light*" OR flares OR "light on vehicles" OR illuminance OR skyglow))) |
| Scopus | (TITLE-ABS-KEY (pollinat* OR "reproductive success" OR "visitation rate*" OR "fruit* set*" OR "seed* set*") AND NOT TITLE-ABS-KEY(freshwater OR aquatic OR fish OR *plankton* OR river OR water OR marine OR sea OR seas OR algorithm OR allerg* OR (bird* AND nest*)))  AND  ((TITLE-ABS-KEY ("light pollut*" OR "artificial light at night" OR ALAN OR ((ultra-violet OR ultraviolet OR UV) AND pollution) OR "street light*" OR streetlight* OR "lighted building*" OR "fishing boat* light*" OR "security light*" OR flares OR "light on vehicles" OR illuminance OR skyglow))  OR  (TITLE(noise OR sound*) OR TITLE-ABS-KEY ("masking auditory" OR "man-made noise" OR "anthropogenic noise" OR "man-made sound*" OR "music festival*") OR ((pollution OR transportation OR road* OR highway* OR motorway* OR railway* OR traffic OR urban OR city OR cities OR construction OR ship* OR boat* OR port* OR aircraft* OR airplane* OR airport* OR industr* OR machinery OR "gas extraction" OR mining OR drilling OR pile-driving OR "communication network*" OR "wind farm*" OR agric* OR farming OR military OR gun* OR visitor*) AND noise) AND NOT TITLE-ABS-KEY ("ultra-sound"))  OR  (TITLE-ABS-KEY ("air contamin*" OR "air pollutant*" OR "air pollution" OR "air quality" OR "atmospheric contamin*" OR "atmospheric pollut*" OR "diesel exhaust" OR (dust OR dusts) OR "exhaust part*" OR "gaseous pollut*" OR "industrial pollut*" OR "industry pollut*" OR ozone OR O3 OR (particle* AND pollut*) OR particulate* OR "particulate matter" OR "persistent organic pollut*" OR "PM2,5" OR "PM 2,5" OR "PM2.5" OR "PM 2.5" OR "PM 10" OR PM10OR POPS OR "road* pollut*" OR "air pollut*" OR smog OR traffic OR "ultrafine particle*" OR "vehicle emission*" OR (VOCS AND pollut*) OR ("Volatile Organic Compounds" AND pollut*) OR (VOC AND pollut*) OR NH3 OR ammonia OR "NOx" OR "nitrogen oxid*" OR "nitrogen dioxid*" OR "sulphur dioxid*" OR benzene OR "Pb" OR "carbon monoxide" OR Benzo*pyrene OR Nickel OR Ni OR cadmium OR Cd OR Arsenic OR "1.3-butadiene" OR "1,3-butadiene" OR "gaseous pollut*" OR "acid rain*") AND NOT TITLE-ABS-KEY ("particle swarm" OR algorithm OR allerg* OR neonicotinoid*))) |
| CAB Abstracts | (pollinat* OR "reproductive success" OR "visitation rate*" OR "fruit* set*" OR "seed* set*") NOT (freshwater OR aquatic OR fish OR *plankton* OR river OR water OR marine OR sea OR seas OR algorithm OR allerg* OR (bird* AND nest*))  AND  ((("light pollut*" OR "artificial light at night" OR ALAN OR ((ultra-violet OR ultraviolet OR UV) AND pollution) OR "street light*" OR streetlight* OR "lighted building*" OR "fishing boat* light*" OR "security light*" OR flares OR "light on vehicles" OR illuminance OR skyglow))  OR  ((noise OR sound*) OR ("masking auditory" OR "man-made noise" OR "anthropogenic noise" OR "man-made sound*" OR "music festival*") OR ((pollution OR transportation OR road* OR highway* OR motorway* OR railway* OR traffic OR urban OR city OR cities OR construction OR ship* OR boat* OR port* OR aircraft* OR airplane* OR airport* OR industr* OR machinery OR "gas extraction" OR mining OR drilling OR pile-driving OR "communication network*" OR "wind farm*" OR agric* OR farming OR military OR gun* OR visitor*) AND noise) NOT ("ultra-sound"))  OR  (("air contamin*" OR "air pollutant*" OR "air pollution" OR "air quality" OR "atmospheric contamin*" OR "atmospheric pollut*" OR "diesel exhaust" OR (dust OR dusts) OR "exhaust part*" OR "gaseous pollut*" OR "industrial pollut*" OR "industry pollut*" OR ozone OR O3 OR (particle* AND pollut*) OR particulate* OR "particulate matter" OR "persistent organic pollut*" OR "PM2,5" OR "PM 2,5" OR "PM2.5" OR "PM 2.5" OR "PM 10" OR PM10OR POPS OR "road* pollut*" OR "air pollut*" OR smog OR traffic OR "ultrafine particle*" OR "vehicle emission*" OR (VOCS AND pollut*) OR ("Volatile Organic Compounds" AND pollut*) OR (VOC AND pollut*) OR NH3 OR ammonia OR "NOx" OR "nitrogen oxid*" OR "nitrogen dioxid*" OR "sulphur dioxid*" OR benzene OR "Pb" OR "carbon monoxide" OR Benzo*pyrene OR Nickel OR Ni OR cadmium OR Cd OR Arsenic OR "1.3-butadiene" OR "1,3-butadiene" OR "gaseous pollut*" OR "acid rain*") NOT ("particle swarm" OR algorithm OR allerg* OR neonicotinoid*))) |

# 3. Results

## 3.1 Extent of the knowledge

### Appendix S4. Studies pertaining to each type of pollution on the different sections of the pollination system.

|  | 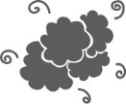  **Air** | 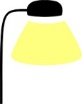  **Light** | **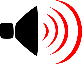**  **Noise** |
| --- | --- | --- | --- |
| Pollinators | (Cook et al., 2020; Dyer, 1999; Leonard et al., 2019b; Liu et al., 2021; Lusebrink et al., 2015; Reitmayer et al., 2022, 2019; Rollin et al., 2022; Ryalls et al., 2022; Thimmegowda et al., 2020; Tommasi et al., 2022; Vanderplanck et al., 2021b) | (Altermatt and Ebert, 2016; Briolat et al., 2021; Deora et al., 2021; Firebaugh and Haynes, 2016; Knop et al., 2017; MacGregor et al., 2017; Wilson et al., 2021) | (Davis et al., 2018; Phillips et al., 2021) |
| Interactions between plants and pollinators | (Cook et al., 2020; Démares et al., 2022; Dötterl et al., 2016; Dubuisson et al., 2022; Duque et al., 2021a, 2021b; Farré-Armengol et al., 2016; Girling et al., 2013; Leonard et al., 2019a; Reitmayer et al., 2022, 2019; Ryalls et al., 2022; Thimmegowda et al., 2020; Vanderplanck et al., 2021a) | (Collins et al., 1997; Conner and Zangori, 1997; Deora et al., 2021; Dzul-Cauich and Munguía-Rosas, 2022; Feldheim and Conner, 1996; Giavi et al., 2021; Knop et al., 2017; MacGregor et al., 2017; Soteras et al., 2022; Stephanou et al., 2000; Van Langevelde et al., 2017a) | (Dzul-Cauich and Munguía-Rosas, 2022; Francis et al., 2012; Phillips et al., 2021) |
| Plant reproductive success | (Ahmadi et al., 2019; Bergweiler and Manning, 1999; Chauhan et al., 2004; Drogoudi and Ashmore, 2000; Duque et al., 2021b, 2021a; Gillespie et al., 2015; Gimeno et al., 2004; Gupta and Ghouse, 1986; Kumar and Singh, 1985; Linskens et al., 1985; Rollin et al., 2022; Roques et al., 1980; Ryalls et al., 2022; Shannon and Mulchi, 1974; Thwe et al., 2015) | (Bariles et al., 2021; Boom et al., 2020; Conner and Neumeier, 2002; Conner and Zangori, 1997; Demchik and Day, 1996; Dzul-Cauich and Munguía-Rosas, 2022; Feldheim and Conner, 1996; Giavi et al., 2020; Knop et al., 2017; Petropoulou et al., 2001; Stephanou et al., 2000; Wang et al., 2008; Wilson et al., 2021) | (Dzul-Cauich and Munguía-Rosas, 2022) |

3.2 Study designs

### Appendix S5. Study designs

| **Study design** | **Studies included** |
| --- | --- |
| Cross-sectional | (Ahmadi et al., 2019; Chauhan et al., 2004; Dzul-Cauich and Munguía-Rosas, 2022; MacGregor et al., 2017; Rollin et al., 2022; Roques et al., 1980; Soteras et al., 2022; Thimmegowda et al., 2020; Tommasi et al., 2022) |
| Experimental | (Altermatt and Ebert, 2016; Bergweiler and Manning, 1999; Collins et al., 1997; Conner and Neumeier, 2002; Cook et al., 2020; Davis et al., 2018; Démares et al., 2022; Demchik and Day, 1996; Deora et al., 2021; Dötterl et al., 2016; Dubuisson et al., 2022; Duque et al., 2021a, 2021b; Farré-Armengol et al., 2016; Feldheim and Conner, 1996; Gillespie et al., 2015; Kumar and Singh, 1985; Leonard et al., 2019a, 2019b; Linskens et al., 1985; Liu et al., 2021; Lusebrink et al., 2015; Reitmayer et al., 2019; Shannon and Mulchi, 1974; Thwe et al., 2015; Van Langevelde et al., 2017a; Vanderplanck et al., 2021a, 2021b) |
| Field experiment | (Bariles et al., 2021; Boom et al., 2020; Conner and Zangori, 1997; Drogoudi and Ashmore, 2000; Firebaugh and Haynes, 2016; Francis et al., 2012; Giavi et al., 2021, 2020; Gimeno et al., 2004; Knop et al., 2017; Petropoulou et al., 2001; Phillips et al., 2021; Reitmayer et al., 2022; Ryalls et al., 2022; Stephanou et al., 2000; Wang et al., 2008; Wilson et al., 2021) |
| Longitudinal | (Gupta and Ghouse, 1986) |
| Modelling | (Briolat et al., 2021; Dyer, 1999) |

### Appendix S6. Habitats investigated by the studies which stated outdoor habitats.

| **Habitats** | **Studies included** |
| --- | --- |
| Grasslands | (Firebaugh and Haynes, 2016; Gimeno et al., 2004; Knop et al., 2017; Wang et al., 2008) |
| Agricultural landscapes | (Ahmadi et al., 2019; Drogoudi and Ashmore, 2000; MacGregor et al., 2017; Phillips et al., 2021; Reitmayer et al., 2022; Rollin et al., 2022; Ryalls et al., 2022) |
| Forests, including scrubland | (Bariles et al., 2021; Boom et al., 2020; Roques et al., 1980; Soteras et al., 2022; Wilson et al., 2021) |
| Urban areas, including gardens, residential areas and roadsides | (Bariles et al., 2021; Chauhan et al., 2004; Conner and Zangori, 1997; Dzul-Cauich and Munguía-Rosas, 2022; Petropoulou et al., 2001; Stephanou et al., 2000; Thimmegowda et al., 2020; Tommasi et al., 2022) |
| Deserts | (Francis et al., 2012) |

## 3.3 Pollution

### Appendix S7. Pollutants. Studies which did not precise the type of pollutants (n=5) were excluded.

| **Category** | **Pollutants** | **Studies included** |
| --- | --- | --- |
| Air pollution  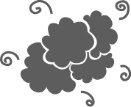 | *Ozone (O_3_)* | (Bergweiler and Manning, 1999; Cook et al., 2020; Démares et al., 2022; Dötterl et al., 2016; Drogoudi and Ashmore, 2000; Dubuisson et al., 2022; Duque et al., 2021a, 2021b; Dyer, 1999; Farré-Armengol et al., 2016; Gillespie et al., 2015; Gimeno et al., 2004; Rollin et al., 2022; Ryalls et al., 2022, 2022; Shannon and Mulchi, 1974; Thwe et al., 2015; Vanderplanck et al., 2021a, 2021b) |
|  | Sulphur dioxide (SO_2_) | (Ahmadi et al., 2019; Kumar and Singh, 1985; Linskens et al., 1985; Roques et al., 1980) |
|  | Diesel exhaust | (Girling et al., 2013; Leonard et al., 2019b; Lusebrink et al., 2015; Reitmayer et al., 2022, 2019; Ryalls et al., 2022) |
|  | Nitric oxides (NO_x_) | (Ahmadi et al., 2019; Girling et al., 2013; Kumar and Singh, 1985; Leonard et al., 2019a; Tommasi et al., 2022) |
|  | Carbon monoxide (CO) | (Ahmadi et al., 2019; Leonard et al., 2019a) |
|  | Particulate matter (PM2.5 and PM10) | (Ahmadi et al., 2019; Liu et al., 2021; Thimmegowda et al., 2020) |
|  | Carbon dioxide (CO_2_) | (Leonard et al., 2019a) |
|  | Coal pollutants | (Gupta and Ghouse, 1986) |
|  | Fluor (F) | (Roques et al., 1980) |
| Light pollution  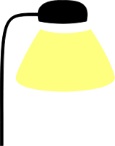 | UV-B | (Collins et al., 1997; Conner and Neumeier, 2002; Conner and Zangori, 1997; Demchik and Day, 1996; Feldheim and Conner, 1996; Petropoulou et al., 2001; Stephanou et al., 2000; Wang et al., 2008) |
|  | LED | (Briolat et al., 2021; Deora et al., 2021; Dzul-Cauich and Munguía-Rosas, 2022; Firebaugh and Haynes, 2016; Giavi et al., 2021, 2020; Knop et al., 2017; Wilson et al., 2021) |
|  | Aerial brightness | (Altermatt and Ebert, 2016; Bariles et al., 2021; Soteras et al., 2022) |
|  | Coloured lights (white, green and red) | (Boom et al., 2020; Van Langevelde et al., 2017a) |
|  | High pressure sodium (HPS) | (Briolat et al., 2021; Dzul-Cauich and Munguía-Rosas, 2022; MacGregor et al., 2017) |
|  | Low pressure sodium (LPS) | (Briolat et al., 2021) |
|  | Mercury vapour | (Briolat et al., 2021) |
|  | Metal halide | (Briolat et al., 2021) |

## 3.4 Pollination

### Appendix S8. Measures of pollination

| **Section** | **Measures of pollination** | **Studies included** |
| --- | --- | --- |
| 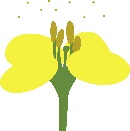Plants reproductive success | Proportion of flowers fruiting | (Ahmadi et al., 2019; Bariles et al., 2021; Bergweiler and Manning, 1999; Boom et al., 2020; Chauhan et al., 2004; Conner and Neumeier, 2002; Conner and Zangori, 1997; Demchik and Day, 1996; Drogoudi and Ashmore, 2000; Duque et al., 2021b; Dzul-Cauich and Munguía-Rosas, 2022; Feldheim and Conner, 1996; Knop et al., 2017; Petropoulou et al., 2001; Ryalls et al., 2022; Thwe et al., 2015; Wang et al., 2008) |
|  | Number of seeds per fruit^[[1]](#footnote-1)^ | (Bariles et al., 2021; Conner and Neumeier, 2002; Conner and Zangori, 1997; Dzul-Cauich and Munguía-Rosas, 2022; Feldheim and Conner, 1996; Giavi et al., 2020; Gillespie et al., 2015; Linskens et al., 1985; Petropoulou et al., 2001; Roques et al., 1980; Ryalls et al., 2022; Wang et al., 2008) |
|  | Number of seeds per plant | (Duque et al., 2021a; Feldheim and Conner, 1996; Petropoulou et al., 2001; Shannon and Mulchi, 1974; Wang et al., 2008) |
|  | Number of fruits per plant | (Drogoudi and Ashmore, 2000; Dzul-Cauich and Munguía-Rosas, 2022; Gupta and Ghouse, 1986; Knop et al., 2017; Kumar and Singh, 1985; Petropoulou et al., 2001; Stephanou et al., 2000; Wilson et al., 2021) |
|  | Mass of seeds per plant | (Gimeno et al., 2004; Ryalls et al., 2022) |
|  | Contribution to crop production | (Rollin et al., 2022) |
| 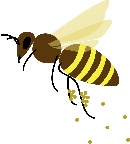Pollinators | Abundance | (Altermatt and Ebert, 2016; Firebaugh and Haynes, 2016; MacGregor et al., 2017; Phillips et al., 2021; Rollin et al., 2022; Ryalls et al., 2022; Wilson et al., 2021) |
|  | Proboscis responses to olfactory cues | (Démares et al., 2022; Leonard et al., 2019a, 2019b; Lusebrink et al., 2015; Reitmayer et al., 2019) |
|  | Species richness | (Knop et al., 2017; MacGregor et al., 2017) |
|  | Colour vision | (Briolat et al., 2021; Dyer, 1999) |
|  | Motility | (Cook et al., 2020; Deora et al., 2021; Liu et al., 2021; Phillips et al., 2021; Reitmayer et al., 2022; Vanderplanck et al., 2021b) |
|  | Morphological traits | (Thimmegowda et al., 2020; Tommasi et al., 2022) |
|  | Physiological responses | (Davis et al., 2018; Reitmayer et al., 2022, 2019; Thimmegowda et al., 2020) |
| 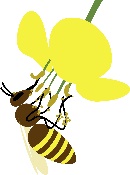Plant-pollinator interactions | Number of pollinator visits | (Conner and Zangori, 1997; Deora et al., 2021; Duque et al., 2021a, 2021b; Dzul-Cauich and Munguía-Rosas, 2022; Farré-Armengol et al., 2016; Francis et al., 2012; Giavi et al., 2021; Knop et al., 2017; Ryalls et al., 2022; Thimmegowda et al., 2020) |
|  | Number of flowers visited | (Conner and Zangori, 1997; Feldheim and Conner, 1996; Phillips et al., 2021; Ryalls et al., 2022; Stephanou et al., 2000) |
|  | Time spent per flower per visit | (Cook et al., 2020; Dzul-Cauich and Munguía-Rosas, 2022; Feldheim and Conner, 1996; Stephanou et al., 2000) |
|  | Pollinators’ feeding behaviour | (Collins et al., 1997; Van Langevelde et al., 2017a; Vanderplanck et al., 2021a) |
|  | Pollen transfer^[[2]](#footnote-2)^ | (Francis et al., 2012; MacGregor et al., 2017; Reitmayer et al., 2022; Soteras et al., 2022) |
|  | Perception of floral volatiles | (Démares et al., 2022; Dötterl et al., 2016) |
|  | Plant volatiles | (Dubuisson et al., 2022; Farré-Armengol et al., 2016; Girling et al., 2013) |

### Appendix S9. Plant species investigated in the different studies and their pollination. Animal pollination does not exclude additional wind pollination. Studies investigating specific habitats for which they might provide species lists were excluded.

| **Type of pollination** | **Clade** | **Family** | **Species** | **English name** | **Studies** |
| --- | --- | --- | --- | --- | --- |
| Wind | Gymnosperms | Pinaceae | *Pinus sylvestris* L. | Scots pine | (Roques et al., 1980) |
|  | Angiosperms | Poaceae | *Triticum aestivum* L. | Wheat | (Shannon and Mulchi, 1974) |
| Animal | Angiosperms | Apocynaceae | *Apocynum androsaemifolium* L. | Spreading dogbane | (Bergweiler and Manning, 1999) |
|  |  |  | *Centaurea* sp. | Centaury | (Giavi et al., 2021) |
|  |  | Apiaceae | *Angelica sylverstris* L. | Wild angelica | (Giavi et al., 2021) |
|  |  |  | *Daucus carota* L. | Wild carrot | (Giavi et al., 2021) |
|  |  |  | *Heracleum sphondylium* L. | Hogweed | (Giavi et al., 2021) |
|  |  | Asparagaceae | *Hesperoyucca whipplei* (Torr.) Trel. | Quixote yucca | (Wilson et al., 2021) |
|  |  | Asteraceae | *Cirsium oleraceum* (L.) Scop. | Cabbage thistle | (Giavi et al., 2021; Knop et al., 2017) |
|  |  |  | *Crepis* sp. | Hawksbeard | (Giavi et al., 2021) |
|  |  |  | *Erigeron annuus* (L.) Pers. | Annual fleabane | (Giavi et al., 2021) |
|  |  |  | *Leontodon* sp. | Kawkbits | (Giavi et al., 2021) |
|  |  | Bigoniaceae | *Tecoma stans* (L.) Juss. ex Kunth | Yellow trumpetbush | (Thimmegowda et al., 2020) |
|  |  | Boraginaceae | *Phacelia campanularia* A. Gray | Desertbells | (Conner and Neumeier, 2002) |
|  |  |  | *Phacelia purshii* Buckl. | Miami mist | (Conner and Neumeier, 2002) |
|  |  | Brassicaceae | *Brassica napus* L. | Rapeseed | (Conner and Zangori, 1997; Demchik and Day, 1996; Feldheim and Conner, 1996; Girling et al., 2013; Rollin et al., 2022) |
|  |  |  | *Brassica nigra* L. | Black mustard | (Conner and Zangori, 1997; Farré-Armengol et al., 2016; Feldheim and Conner, 1996; Ryalls et al., 2022) |
|  |  |  | *Brassica rapus* L. | Field mustard | (Demchik and Day, 1996; Feldheim and Conner, 1996) |
|  |  |  | *Malcolmia maritima* (L.) R. Br. | Virginia stock | (Petropoulou et al., 2001) |
|  |  |  | *Sinapis arvensis* L. | Field mustard | (Duque et al., 2021a, 2021b) |
|  |  | Caprifoliaceae | *Knautia dipsacifolia* Kreuzer | Wood scabious | (Giavi et al., 2021) |
|  |  |  | *Valeriana officinalis* L. | Valerian | (Giavi et al., 2021) |
|  |  | Caryophyllaceae | *Cerastium glomeratum* Thuill. | Sticky mouse-ear chickweed | (Wang et al., 2008) |
|  |  |  | *Silene latifolia* Poir. | White campion | (Boom et al., 2020) |
|  |  | Cistaceae | *Cistus creticus* L. | Pink rock-rose | (Stephanou et al., 2000) |
|  |  | Ericaceae | *Vaccinium corymbosum* L. | Northern highbush blueberry | (Rollin et al., 2022) |
|  |  | Fabaceae | *Cassia siamea* Irwin and Braneby | Cassod tree | (Chauhan et al., 2004) |
|  |  |  | *Cicer arietinum* L. | Chickpea | (Kumar and Singh, 1985) |
|  |  |  | *Erythrostemon gilliesii* (Hook.) Klotzsch | Bird of paradise | (Bariles et al., 2021; Soteras et al., 2022) |
|  |  |  | *Trifolium cherleri* L. | Cupped clover | (Gimeno et al., 2004) |
|  |  |  | *Trifolium pratense* L. | Red clover | (Giavi et al., 2021) |
|  |  |  | *Trifolium striatum* L. | Knotted clover | (Gimeno et al., 2004) |
|  |  |  | *Trifolium subterraneum* L. | Subterranean clover | (Gimeno et al., 2004) |
|  |  |  | *Vicia faba* L. | Broad bean | (Rollin et al., 2022) |
|  |  | Gerianaceae | *Geranium* sp. | Geraniums | (Giavi et al., 2021) |
|  |  |  | *Geranium graveolens* L’Hér. | Rose geranium | (Leonard et al., 2019b) |
|  |  |  | *Geranium molle* L. | Dovesfoot geranium | (Giavi et al., 2021) |
|  |  |  | *Geranium sylvaticum* L. | Wood cranesbill | (Giavi et al., 2021) |
|  |  | Lamiaceae | *Lavandula angustifolia* Mill. | Lavender | (Dubuisson et al., 2022; Leonard et al., 2019a) |
|  |  |  | *Stachys* sp. | Hedgenettle | (Giavi et al., 2021) |
|  |  | Malvaceae | *Abelmoschus esculentus* Moench. | Okra | (Gupta and Ghouse, 1986) |
|  |  |  | *Ceiba peitandra* (L.) Gaertn*.* | Kapok | (Dzul-Cauich and Munguía-Rosas, 2022) |
|  |  |  | *Malus domestica* Borkh. | Apples | (Ahmadi et al., 2019; Rollin et al., 2022) |
|  |  | Onagraceae | *Epilobium* sp. | Willowherbs | (Giavi et al., 2021) |
|  |  |  | *Epilobium angustifolium* L. | Fireweed | (Giavi et al., 2020) |
|  |  | Ranunculaceae | *Ranunculus* sp. | Buttercups | (Giavi et al., 2021) |
|  |  | Rosaceae | *Filipendula ulmaria* (L.) Maxim. | Meadowsweet | (Giavi et al., 2021) |
|  |  |  | *Fragaria x ananassa* Duch. | Strawberries | (Drogoudi and Ashmore, 2000) |
|  |  |  | *Rubus* sp. | Bramble | (Giavi et al., 2021) |
|  |  | Rubiaceae | *Galium* sp. | Bedstraw | (Giavi et al., 2021) |
|  |  | Solanaceae | *Petunia hybrida* Vilm. | Common garden petunia | (Linskens et al., 1985) |
|  |  |  | *Solanum lycoperscicum* Mill. | Tomatoes | (Gillespie et al., 2015; Thwe et al., 2015) |

### Appendix S10. Pollinators studied, as given in each article.

| **Pollinators** | | | | | **Article reference** |
| --- | --- | --- | --- | --- | --- |
| **Class** | **Order** | **Family** | **Species** | **English name** |  |
| Insecta | Hympenoptera |  |  |  | (Knop et al., 2017) |
|  |  | Apideae |  |  | (Duque et al., 2021a; Dyer, 1999; Rollin et al., 2022; Ryalls et al., 2022) |
|  |  |  | *Apis mellifera* | Honeybee | (Collins et al., 1997; Démares et al., 2022; Dötterl et al., 2016; Duque et al., 2021b; Girling et al., 2013; Leonard et al., 2019a, 2019b; Lusebrink et al., 2015; Reitmayer et al., 2022, 2019; Rollin et al., 2022) |
|  |  |  | *Apis dorsata* | Giant honeybee | (Thimmegowda et al., 2020) |
|  |  |  | *Bombus* sp. | Bumblebees | (Duque et al., 2021b; Phillips et al., 2021) |
|  |  |  | *Bombus terrestris* | Buff-tailed bumblebee | (Farré-Armengol et al., 2016; Tommasi et al., 2022; Vanderplanck et al., 2021a) |
|  |  |  | *Bombus pascorum* | Common carder bee | (Tommasi et al., 2022) |
|  |  | Megachilidae | *Osmia* sp. | Mason bees | (Duque et al., 2021b) |
|  |  | Agaonideae | *Blastophaga psenes* | Fig wasps | (Vanderplanck et al., 2021a, 2021b) |
|  | Lepidoptera |  |  |  | (Knop et al., 2017) |
|  |  |  |  | Moths | (MacGregor et al., 2017; Ryalls et al., 2022) |
|  |  | Erebidae | *Rivula sericealis* | Straw dot | (Van Langevelde et al., 2017b) |
|  |  | Geometridae | *Dysstroma truncata* | Common marbled carpet | (Van Langevelde et al., 2017b) |
|  |  |  | *Idaea biselata* | Small fan-footed wave | (Van Langevelde et al., 2017b) |
|  |  | Nymphalidae | *Danaus plexippus* | Monarch butterfly | (Davis et al., 2018) |
|  |  |  | *Vanessa cardui* | Painted lady | (Liu et al., 2021) |
|  |  | Prodoxidae | *Tegeticula maculate* | Yucca moth | (Wilson et al., 2021) |
|  |  | Sphingidae | *Manduca sexta* | Tobacco hawkmoth | (Cook et al., 2020; Deora et al., 2021) |
|  |  |  | *Deilephila elpenor* | Elephant hawkmoth | (Briolat et al., 2021) |
|  |  | Yponomeutidae | *Yponomeuta cagnagella* | Spindle ermine | (Firebaugh and Haynes, 2016) |
| Insecta | Diptera |  |  |  | (Knop et al., 2017) |
|  |  | Syrphidae |  | Hoverflies | (Duque et al., 2021b, 2021a; Rollin et al., 2022; Ryalls et al., 2022) |
|  |  |  | *Photinus pyralis* | Common eastern firefly | (Firebaugh and Haynes, 2016) |
|  | Coleoptera |  |  |  | (Knop et al., 2017) |
|  |  | Lampyridae | *Photuris versicolor* |  | (Firebaugh and Haynes, 2016) |
| Aves | Apodiformes | Trochilidae | *Archilochus alexandri* | Black-chinned hummingbird | (Francis et al., 2012) |

## 3.5 Impact of pollution

### Appendix S11. Parameter estimates, standard error, z-values, p-values and the confidence interval (95%) for the single best-fit model (DeltaAICc≤2) for air pollution.

|  |  | **Estimate** | **SE** | **z-value** | **p-value** | **Confidence interval** |
| --- | --- | --- | --- | --- | --- | --- |
| Intercept | | -0.943 | 0.221 | -4.271 | <.001 *** | -1.3763, -0.5104 |
| Study type | |  |  |  |  |  |
|  | Experimental | 0.472 | 0.207 | 2.284 | 0.0224 * | 0.0669, 0.877 |
|  | Field experiment | 0.685 | 0.217 | 3.160 | 0.0016 ** | 0.2601, 1.1098 |
|  | Longitudinal | -0.118 | 0.604 | -0.195 | 0.8453 | -1.3017, 1.066 |
|  | Modelling | -3.058 | 0.574 | -5.331 | <.001 *** | -4.1825, -1.9337 |
| Section of the pollination system | | | | |  |  |
|  | Plant reproductive success | -0.063 | 0.172 | -0.365 | 0.7149 | -0.4006, 0.2747 |
|  | Pollinators | 0.430 | 0.180 | 2.393 | 0.0167 * | 0.0778, 0.7818 |


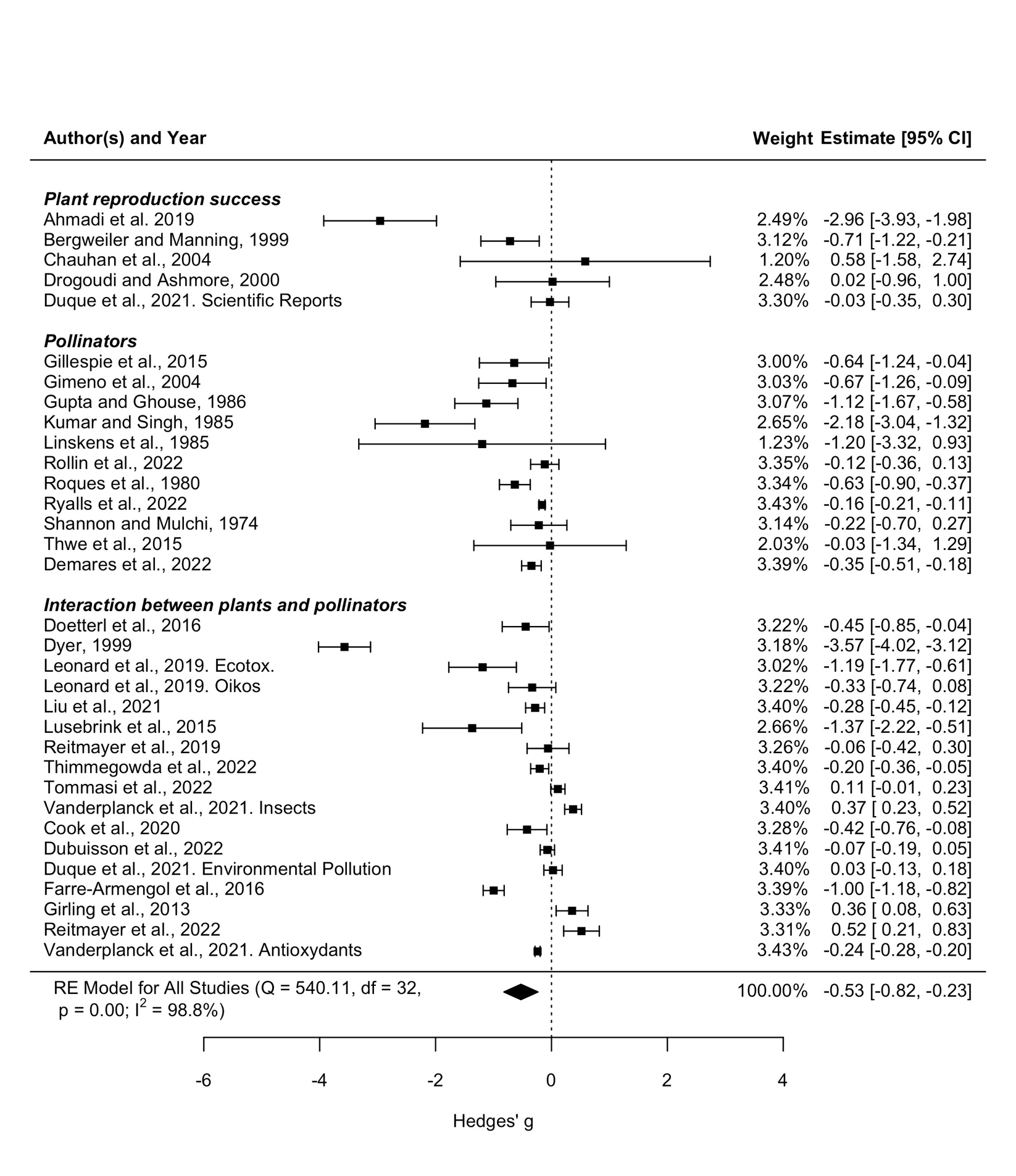


### **Appendix S12. Forest plot of all included studies on the impact of air pollution on pollination**, **including the results of the model without explanatory variables.** Studies are considered to have a significant impact if the error-bar does not cross the 0 axis. Negative Hedges’ g values indicate a negative impact of pollution on pollination, positive Hedges’ g values a positive impact.

### Appendix S13. Parameter estimates, standard error, z-values and p-values and the confidence interval (95%) for the best-fit models (DeltaAICc≤2) for light pollution.

|  | **Estimate** | **Std. Error** | **z-value** | **p-value** | **Confidence interval** |
| --- | --- | --- | --- | --- | --- |
| (Intercept) | 0.288 | 0.306 | 0.943 | 0.346 | -0.311, 0.887 |


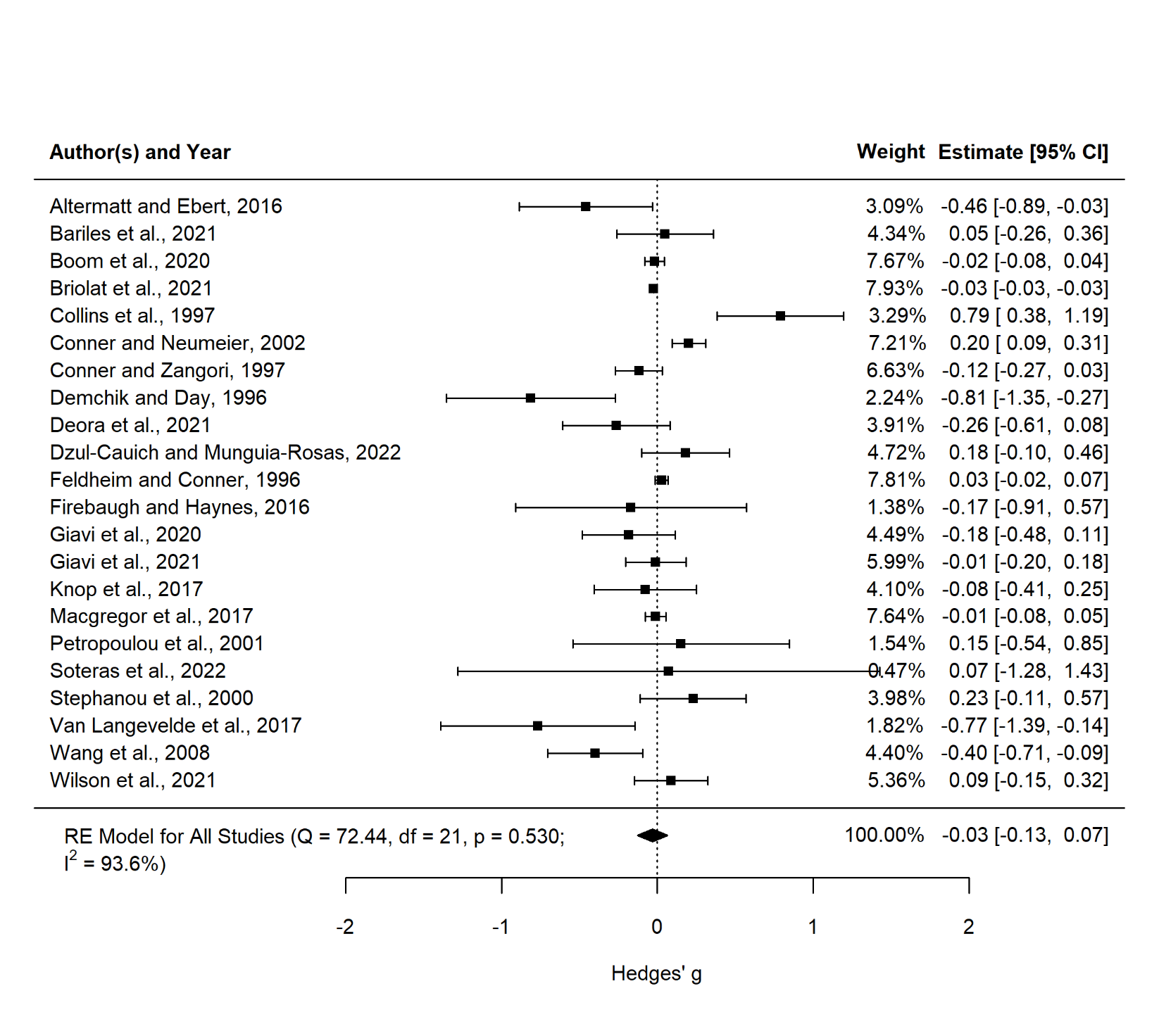


### **Appendix S14. Forest plot of all included studies on the impact of light pollution on pollination**, including the results of the model without explanatory variables. Studies are considered to have a significant impact if the error-bar does not cross the 0 axis. Negative Hedges’ g values indicate a negative impact of pollution on pollination, positive Hedges’ g values a positive impact.

### Appendix S15. Parameter estimates, standard error, z-values and p-values and the confidence interval (95%) for the best-fit models (DeltaAICc≤2) for noise pollution.

|  | **Estimate** | **Std. Error** | **z-value** | **p-value** | **Confidence interval** |
| --- | --- | --- | --- | --- | --- |
| (Intercept) | 0.247 | 0.153 | 1.615 | 0.106 | -0.053, 0.542 |


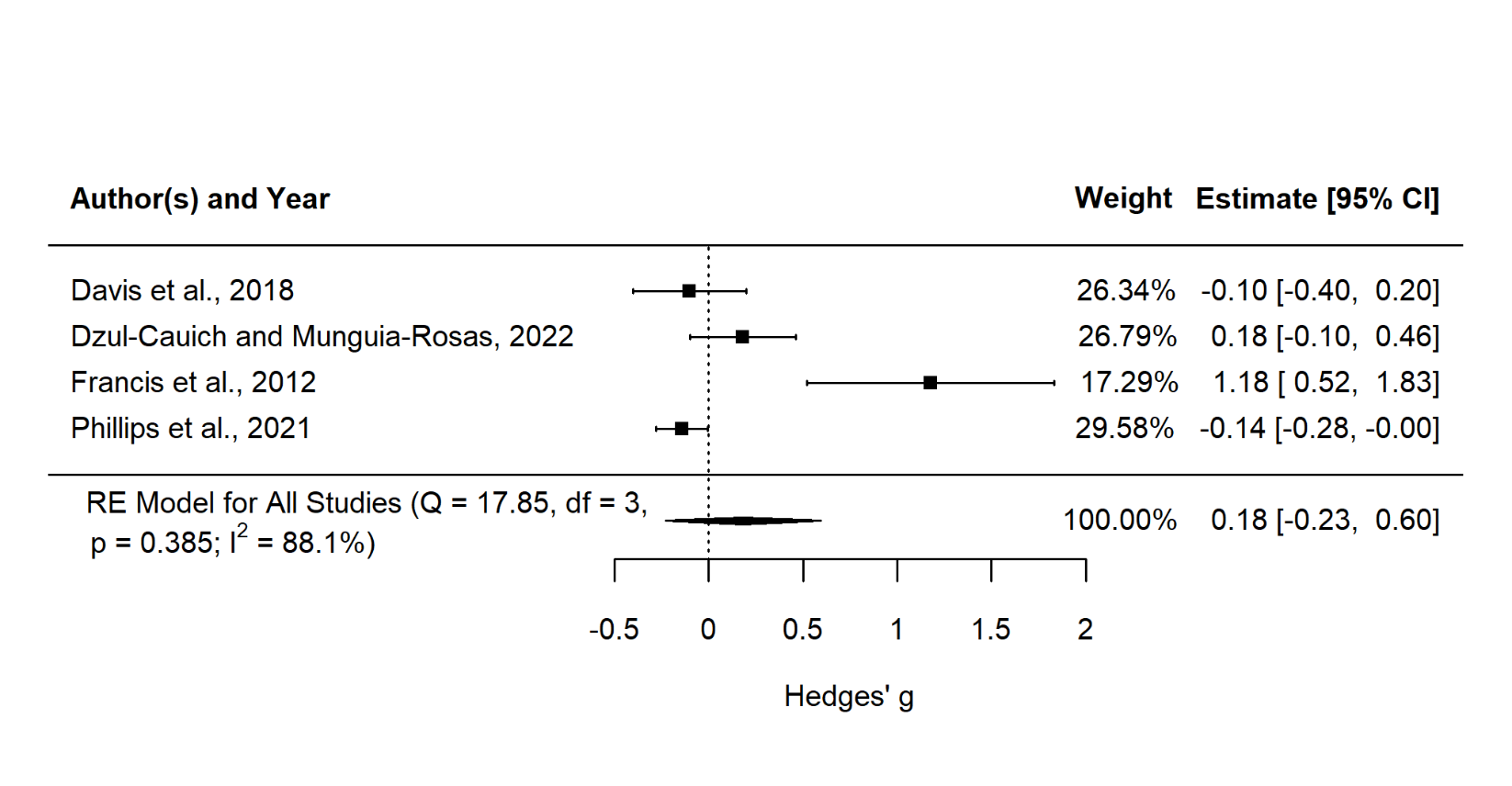


### **Appendix S16. Forest plot of all included studies on the impact of noise pollution on pollination**, including the results of the model without explanatory variables. Studies are considered to have a significant impact if the error-bar does not cross the 0 axis. Negative Hedges’ g values indicate a negative impact of pollution on pollination, positive Hedges’ g values a positive impact.

# References

Ahmadi, F., ValizadehKaji, B., Abbasifar, A., 2019. The effect of air pollution on developmental stages and pollen germination, pollen tube growth and fruit Set of the apple cultivars ‘Golden Delicious’ and ‘Golab.’ Gesunde Pflanzen 71, 237–247. https://doi.org/10.1007/s10343-019-00468-4

Altermatt, F., Ebert, D., 2016. Reduced flight-to-light behaviour of moth populations exposed to long-term urban light pollution. Biology Letters 12, 20160111. https://doi.org/10.1098/rsbl.2016.0111

Bariles, J.B., Cocucci, A.A., Soteras, F., 2021. Pollination and fitness of a hawkmoth-pollinated plant are related to light pollution and tree cover. Biological Journal of the Linnean Society 134, 815–822. https://doi.org/10.1093/biolinnean/blab114

Bergweiler, C.J., Manning, W.J., 1999. Inhibition of flowering and reproductive success in spreading dogbane (Apocynum androsaemifolium) by exposure to ambient ozone. Environmental Pollution 105, 333–339. https://doi.org/10.1016/S0269-7491(99)00044-5

Boom, M.P., Spoelstra, K., Biere, A., Knop, E., Visser, M.E., 2020. Pollination and fruit infestation under artificial light at night:light colour matters. Sci Rep 10, 18389. https://doi.org/10.1038/s41598-020-75471-1

Briolat, E.S., Gaston, K.J., Bennie, J., Rosenfeld, E.J., Troscianko, J., 2021. Artificial nighttime lighting impacts visual ecology links between flowers, pollinators and predators. Nat Commun 12, 4163.

Chauhan, S.V.S., Chaurasia, B., Rana, A., 2004. Impact of air pollution on floral morphology of Cassia siamea Lamk. Journal of Environmental Biology 25, 291–297.

Collins, S.A., Conner, J.K., Robinson, G.E., 1997. Foraging behavior of honey bees (Hymenoptera: Apidae) on Brassica nigra and B. rapa grown under simulated ambient and enhanced UV-B radiation. Annals of the Entomological Society of America 90, 5.

Conner, J.K., Neumeier, R., 2002. The effects of ultraviolet-B radiation and intraspecific competition on growth, pollination success, and lifetime female fitness in Phacelia campanularia and P. purshii (Hydrophyllaceae). American Journal of Botany 89, 103–110. https://doi.org/10.3732/ajb.89.1.103

Conner, J.K., Zangori, L.A., 1997. A garden study of the effects of ultraviolet-B radiation on pollination success and lifetime female fitness in Brassica. Oecologia 111, 388–395. https://doi.org/10.1007/s004420050250

Cook, B., Haverkamp, A., Hansson, B.S., Roulston, T., Lerdau, M., Knaden, M., 2020. Pollination in the Anthropocene: a moth can learn ozone-altered floral blends. J Chem Ecol 46, 987–996. https://doi.org/10.1007/s10886-020-01211-4

Curti, S., Gori, D., Di Gregori, V., Farioli, A., Baldasseroni, A., Fantini, M.P., Christiani, D.C., Violante, F.S., Mattioli, S., 2016. PubMed search filters for the study of putative outdoor air pollution determinants of disease. BMJ Open 6. https://doi.org/10.1136/bmjopen-2016-013092

Davis, A.K., Schroeder, H., Yeager, I., Pearce, J., 2018. Effects of simulated highway noise on heart rates of larval monarch butterflies, Danaus plexippus : implications for roadside habitat suitability. Biology Letters 14, 20180018. https://doi.org/10.1098/rsbl.2018.0018

Defra, 2019. Clean air strategy. https://doi.org/10.5694/j.1326-5377.1962.tb59377.x

Démares, F., Gibert, L., Creusot, P., Lapeyre, B., Proffit, M., 2022. Acute ozone exposure impairs detection of floral odor, learning, and memory of honey bees, through olfactory generalization. Sci Total Environ 827, 154342. https://doi.org/10.1016/j.scitotenv.2022.154342

Demchik, S.M., Day, T.A., 1996. Effect of enhanced UV-B radiation on pollen quantity, quality, and seed yield in Brassica rapa (Brassicaceae) . American Journal of Botany 83, 573–579. https://doi.org/10.1002/j.1537-2197.1996.tb12741.x

Deora, T., Ahmed, M.A., Brunton, B.W., Daniel, T.L., 2021. Learning to feed in the dark: how light level influences feeding in the hawkmoth Manduca sexta. Biology Letters 17, 20210320. https://doi.org/10.1098/rsbl.2021.0320

Dötterl, S., Vater, M., Rupp, T., Held, A., 2016. Ozone Differentially Affects Perception of Plant Volatiles in Western Honey Bees. Journal of Chemical Ecology 42, 486–489. https://doi.org/10.1007/s10886-016-0717-8

Drogoudi, P.D., Ashmore, M.R., 2000. Does elevated ozone have differing effects in flowering and deblossomed strawberry? New Phytologist 147, 561–569. https://doi.org/10.1046/j.1469-8137.2000.00718.x

Dubuisson, C., Nicolè, F., Buatois, B., Hossaert-McKey, M., Proffit, M., 2022. Tropospheric Ozone Alters the Chemical Signal Emitted by an Emblematic Plant of the Mediterranean Region: The True Lavender (Lavandula angustifolia Mill.). Frontiers in Ecology and Evolution 10.

Duque, L., Poelman, E.H., Steffan-Dewenter, I., 2021a. Plant age at the time of ozone exposure affects flowering patterns, biotic interactions and reproduction of wild mustard. Sci Rep 11, 23448. https://doi.org/10.1038/s41598-021-02878-9

Duque, L., Poelman, E.H., Steffan-Dewenter, I., 2021b. Effects of ozone stress on flowering phenology, plant-pollinator interactions and plant reproductive success. Environmental Pollution 272, 115953. https://doi.org/10.1016/j.envpol.2020.115953

Dyer, A.G., 1999. Atmospheric ozone concentration and the colour vision of insect pollinators. Australian Journal of Zoology 47, 529–538.

Dzul-Cauich, H.F., Munguía-Rosas, M.A., 2022. Negative effects of light pollution on pollinator visits are outweighed by positive effects on the reproductive success of a bat-pollinated tree. Sci Nat 109, 12. https://doi.org/10.1007/s00114-021-01783-5

Farré-Armengol, G., Peñuelas, J., Li, T., Yli-Pirilä, P., Filella, I., Llusia, J., Blande, J.D., 2016. Ozone degrades floral scent and reduces pollinator attraction to flowers. New Phytologist 209, 152–160. https://doi.org/10.1111/nph.13620

Feldheim, K., Conner, J.K., 1996. The effects of increased UV-B radiation on growth, pollination success, and lifetime female fitness in two Brassica species. Oecologia 106, 284–297. https://doi.org/10.1007/BF00334556

Firebaugh, A., Haynes, K.J., 2016. Experimental tests of light-pollution impacts on nocturnal insect courtship and dispersal. Oecologia 182, 1203–1211. https://doi.org/10.1007/s00442-016-3723-1

Francis, C.D., Kleist, N.J., Ortega, C.P., Cruz, A., 2012. Noise pollution alters ecological services: enhanced pollination and disrupted seed dispersal. Proceedings of the Royal Society B: Biological Sciences 279, 2727–2735. https://doi.org/10.1098/rspb.2012.0230

Giavi, S., Blösch, S., Schuster, G., Knop, E., 2020. Artificial light at night can modify ecosystem functioning beyond the lit area. Sci Rep 10, 11870. https://doi.org/10.1038/s41598-020-68667-y

Giavi, S., Fontaine, C., Knop, E., 2021. Impact of artificial light at night on diurnal plant-pollinator interactions. Nature Communications 12, 1690. https://doi.org/10.1038/s41467-021-22011-8

Gillespie, C., Stabler, D., Tallentire, E., Goumenaki, E., Barnes, J., 2015. Exposure to environmentally-relevant levels of ozone negatively influence pollen and fruit development. Environmental Pollution 206, 494–501. https://doi.org/10.1016/j.envpol.2015.08.003

Gimeno, B.S., Bermejo, V., Sanz, J., de la Torre, D., Gil, J.M., 2004. Assessment of the effects of ozone exposure and plant competition on the reproductive ability of three therophytic clover species from Iberian pastures. Atmospheric Environment 38, 2295–2303. https://doi.org/10.1016/j.atmosenv.2003.10.062

Girling, R.D., Lusebrink, I., Farthing, E., Newman, T.A., Poppy, G.M., 2013. Diesel exhaust rapidly degrades floral odours used by honeybees. Scientific Reports 3, 2779. https://doi.org/10.1038/srep02779

Gupta, M.C., Ghouse, A.K.M., 1986. Fruit quality and yield of Abeloschus esculentus Moench in relation to air pollution. Acta botanica Indica 14, 191–194.

Haddaway, N.R., Page, M.J., Pritchard, C.C., McGuinness, L.A., 2022. PRISMA2020: An R package and Shiny app for producing PRISMA 2020-compliant flow diagrams, with interactivity for optimised digital transparency and Open Synthesis. Campbell Systematic Reviews 18, e1230. https://doi.org/10.1002/cl2.1230

Knop, E., Zoller, L., Ryser, R., Gerpe, C., Hörler, M., Fontaine, C., 2017. Artificial light at night as a new threat to pollination. Nature 548, 206–209. https://doi.org/10.1038/nature23288

Kumar, N., Singh, V., 1985. Effect of SO2 and NO2 pollution on Cicer arietinum. Indian Journal of Ecology 12, 183–188.

Leonard, R.J., Pettit, T.J., Irga, P., McArthur, C., Hochuli, D.F., 2019a. Acute exposure to urban air pollution impairs olfactory learning and memory in honeybees. Ecotoxicology 28, 1056–1062. https://doi.org/10.1007/s10646-019-02081-7

Leonard, R.J., Vergoz, V., Proschogo, N., McArthur, C., Hochuli, D.F., 2019b. Petrol exhaust pollution impairs honey bee learning and memory. Oikos 128, 264–273. https://doi.org/10.1111/oik.05405

Linskens, H.F., van Megen, Y., Pfahler, P.L., Wilcox, M., 1985. Sulfur dioxide effects on petunia pollen germination and seed set. Bulletin of Environmental Contamination and Toxicology 34, 691–695. https://doi.org/10.1007/BF01609794

Liu, Y., Wooster, M.J., Grosvenor, M.J., Lim, K.S., Francis, R.A., 2021. Strong impacts of smoke polluted air demonstrated on the flight behaviour of the painted lady butterfly (Vanessa cardui L.). Ecological Entomology 46, 195–208. https://doi.org/10.1111/een.12952

Longcore, T., Rich, C., 2004. Ecological light pollution. Frontiers in Ecology and the Environment 2, 191–198. https://doi.org/10.1890/1540-9295(2004)002[0191:ELP]2.0.CO;2

Lusebrink, I., Girling, R.D., Farthing, E., Newman, T.A., Jackson, C.W., Poppy, G.M., 2015. The effects of diesel exhaust pollution on floral volatiles and the consequences for honey bee olfaction. Journal of Chemical Ecology 41, 904–912. https://doi.org/10.1007/s10886-015-0624-4

MacGregor, C.J., Evans, D.M., Fox, R., Pocock, M.J.O., 2017. The dark side of street lighting: impacts on moths and evidence for the disruption of nocturnal pollen transport. Global Change Biology 23, 697–707. https://doi.org/10.1111/gcb.13371

Petropoulou, Y., Georgiou, O., Psaras, G.K., Manetas, Y., 2001. Improved flower advertisement, pollinator rewards and seed yield by enhanced UV-B radiation in the Mediterranean annual Malcolmia maritima. New Phytologist 152, 85–90. https://doi.org/10.1046/j.0028-646x.2001.00241.x

Phillips, B.B., Bullock, J.M., Gaston, K.J., Hudson‐Edwards, K.A., Bamford, M., Cruse, D., Dicks, L.V., Falagan, C., Wallace, C., Osborne, J.L., 2021. Impacts of multiple pollutants on pollinator activity in road verges. Journal of Applied Ecology 1365-2664.13844. https://doi.org/10.1111/1365-2664.13844

Reitmayer, C.M., Girling, R.D., Jackson, C.W., Newman, T.A., 2022. Repeated short-term exposure to diesel exhaust reduces honey bee colony fitness. Environmental Pollution 300, 118934. https://doi.org/10.1016/j.envpol.2022.118934

Reitmayer, C.M., Ryalls, J.M.W., Farthing, E., Jackson, C.W., Girling, R.D., Newman, T.A., 2019. Acute exposure to diesel exhaust induces central nervous system stress and altered learning and memory in honey bees. Scientific Reports 9, 1–9. https://doi.org/10.1038/s41598-019-41876-w

Rollin, O., Aguirre‐Gutiérrez, J., Yasrebi-de Kom, I.A.R., Garratt, M.P.D., de Groot, G.A., Kleijn, D., Potts, S.G., Scheper, J., Carvalheiro, L.G., 2022. Effects of ozone air pollution on crop pollinators and pollination. Global Environmental Change 75, 102529. https://doi.org/10.1016/j.gloenvcha.2022.102529

Roques, A., Kerjean, M., Auclair, D., 1980. Effets de la pollution atmospherique par le fluor et le dioxyde de soufre sur l’appareil reproducteur femelle de Pinus silvestris en foret de roumare (Seine-Maritime, France). Environmental Pollution. Series A, Ecological and Biological 21, 191–201. https://doi.org/10.1016/0143-1471(80)90163-4

Ryalls, J.M.W., Langford, B., Mullinger, N.J., Bromfield, L.M., Nemitz, E., Pfrang, C., Girling, R.D., 2022. Anthropogenic air pollutants reduce insect-mediated pollination services. Environmental Pollution 297, 118847. https://doi.org/10.1016/j.envpol.2022.118847

Shannon, J.G., Mulchi, C.L., 1974. Ozone damage to wheat varieties at anthesis 1. Crop Science 14, 335–337. https://doi.org/10.2135/cropsci1974.0011183x001400020052x

Sordello, R., Flamerie De Lachapelle, F., Livoreil, B., Vanpeene, S., 2019. Evidence of the environmental impact of noise pollution on biodiversity: a systematic map protocol. Environmental Evidence 8, 8. https://doi.org/10.1186/s13750-019-0146-6

Soteras, F., Camps, G.A., Costas, S.M., Giaquinta, A., Peralta, G., Cocucci, A.A., 2022. Fragility of nocturnal interactions: Pollination intensity increases with distance to light pollution sources but decreases with increasing environmental suitability. Environmental Pollution 292, 118350. https://doi.org/10.1016/j.envpol.2021.118350

Stephanou, M., Petropoulou, Y., Georgiou, O., Manetas, Y., 2000. Enhanced UV-B radiation, flower attributes and pollinator behaviour in Cistus creticus: A Mediterranean field study. Plant Ecology 147, 165–171. https://doi.org/10.1023/A:1009802401955

Thimmegowda, G.G., Mullen, S., Sottilare, K., Sharma, A., Soham Mohanta, S., Brockmann, A., Dhandapany, P.S., Olsson, S.B., 2020. A field-based quantitative analysis of sublethal effects of air pollution on pollinators. PNAS 117, 20653–20661. https://doi.org/10.1073/pnas.2009074117

Thwe, A.A., Vercambre, G., Gautier, H., Gay, F., Phattaralerphong, J., Kasemsap, P., 2015. Effects of acute ozone stress on reproductive traits of tomato, fruit yield and fruit composition. Journal of the Science of Food and Agriculture 95, 614–620. https://doi.org/10.1002/jsfa.6798

Tommasi, N., Pioltelli, E., Biella, P., Labra, M., Casiraghi, M., Galimberti, A., 2022. Effect of urbanization and its environmental stressors on the intraspecific variation of flight functional traits in two bumblebee species. Oecologia 199, 289–299. https://doi.org/10.1007/s00442-022-05184-x

Van Langevelde, F., Van Grunsven, R.H.A.A., Veenendaal, E.M., Fijen, T.P.M.M., 2017a. Artificial night lighting inhibits feeding in moths. Biology Letters 13, 2–5. https://doi.org/10.1098/rsbl.2016.0874

Van Langevelde, F., Van Grunsven, R.H.A.A., Veenendaal, E.M., Fijen, T.P.M.M., 2017b. Artificial night lighting inhibits feeding in moths. Biology Letters 13, 2–5. https://doi.org/10.1098/rsbl.2016.0874

Vanderplanck, M., Lapeyre, B., Brondani, M., Opsommer, M., Dufay, M., Hossaert-McKey, M., Proffit, M., 2021a. Ozone Pollution Alters Olfaction and Behavior of Pollinators. Antioxidants 10, 636. https://doi.org/10.3390/antiox10050636

Vanderplanck, M., Lapeyre, B., Lucas, S., Proffit, M., 2021b. Ozone Induces Distress Behaviors in Fig Wasps with a Reduced Chance of Recovery. Insects 12, 995. https://doi.org/10.3390/insects12110995

Wang, Y., Qiu, N., Wang, X., Ma, Z., Du, G., 2008. Effects of enhanced UV-B radiation on fitness of an alpine species Cerastium glomeratum Thuill. Journal of Plant Ecology 1, 197–202. https://doi.org/10.1093/jpe/rtn018

Wilson, A.A., Seymoure, B.M., Jaeger, S., Milstead, B., Payne, H., Peria, L., Vosbigian, R.A., Francis, C.D., 2021. Direct and Ambient Light Pollution Alters Recruitment for a Diurnal Plant–Pollinator System. Integrative and Comparative Biology 61, 1122–1133. https://doi.org/10.1093/icb/icab010

1. For synthesising purposes, gymnosperm’s cones and Poaceae’s kernels were considered the equivalent of fruits [↑](#footnote-ref-1)
2. Includes the probability of carrying pollen, the number of pollinated flowers, the number of plants with pollen, and pollen richness and count. [↑](#footnote-ref-2)
